# Aldo‐keto reductase enzymes detoxify glyphosate and improve herbicide resistance in plants

**DOI:** 10.1111/pbi.12632

**Published:** 2017-05-11

**Authors:** Ramu S. Vemanna, Amaranatha Reddy Vennapusa, Murugesh Easwaran, Babitha K. Chandrashekar, Hanumantha Rao, Kirankumar Ghanti, Chinta Sudhakar, Kirankumar S. Mysore, Udayakumar Makarla

**Affiliations:** ^1^ Department of Crop PhysiologyUniversity of Agricultural SciencesGKVKBangaloreIndia; ^2^ Plant Biology DivisionThe Samuel Roberts Noble FoundationArdmoreOKUSA; ^3^ Department of BotanySri Krishnadevaraya UniversityAnantapurIndia; ^4^ Department of BioinformaticsCentre for BioinformaticsBharathiar UniversityCoimbatoreIndia; ^5^ Present address: Orris Life SciencesBangaloreIndia; ^6^ Present address: Monsanto Research CenterBangaloreIndia

**Keywords:** herbicide tolerance, aldo‐keto reductase, glyphosate, residual toxicity, transgenic plants, shikimic acid, photosynthesis, protein docking, GM plants

## Abstract

In recent years, concerns about the use of glyphosate‐resistant crops have increased because of glyphosate residual levels in plants and development of herbicide‐resistant weeds. In spite of identifying glyphosate‐detoxifying genes from microorganisms, the plant mechanism to detoxify glyphosate has not been studied. We characterized an aldo‐keto reductase gene from *Pseudomonas* (*PsAKR1*) and rice (*OsAKR1*) and showed, by docking studies, both PsAKR1 and OsAKR1 can efficiently bind to glyphosate. Silencing *AKR1* homologues in rice and *Nicotiana benthamiana* or mutation of *AKR1* in yeast and *Arabidopsis* showed increased sensitivity to glyphosate. External application of AKR proteins rescued glyphosate‐mediated cucumber seedling growth inhibition. Regeneration of tobacco transgenic lines expressing *PsAKR1* or *OsAKRI* on glyphosate suggests that *AKR* can be used as selectable marker to develop transgenic crops. *PsAKR1‐* or *OsAKRI*‐expressing tobacco and rice transgenic plants showed improved tolerance to glyphosate with reduced accumulation of shikimic acid without affecting the normal photosynthetic rates. These results suggested that *AKR1* when overexpressed detoxifies glyphosate *in planta*.

## Introduction

Plant productivity is reduced by many environmental stresses including biotic and abiotic factors. The reduction in yield due to competition with weeds is higher than other factors (Farkas, 2006). Weeds compete with crops for water, nutrients, sunlight and space. In addition, weed seeds contaminate crop harvests and reduce grain quality. Weed management is an important agricultural practice, and many chemicals have been identified and used as selective/nonselective herbicides for effective control of a wide range of weeds (Kohler and Triebskorn, 2013). Glyphosate is one of the most widely used herbicides because of its low cost, low toxicity and effective against broad spectrum of weeds, but it is nonselective (Green and Owen, 2011). Several crops have been genetically modified to tolerate nonselective herbicides. Soya bean, cotton, maize, alfalfa, turfgrass and canola have been engineered to tolerate glyphosate (Green, 2012). Globally, in 2013 alone, 99.4 million hectares or 57 per cent of the 175.2 million hectares that grow genetically modified crops were planted with herbicide‐tolerant crops. The most common are glyphosate‐ and glufosinate‐tolerant crops (http://www.isaaa.org/gmapprovaldatabase).

The introduction of glyphosate‐tolerant crops transformed the way many growers manage weeds. Glyphosate inhibits the activity of a nuclear‐encoded, plastid‐localized enzyme, 5‐enolpyruvylshikimate‐3‐phosphate synthase (EPSPS), which is involved in the shikimic acid pathway of plants and microorganisms (Schonbrunn *et al*., 2001; Sost and Amerhein, 1990). In 1983, scientists at Monsanto and Washington University isolated the common soil bacteria, *Agrobacterium tumefaciens* strain CP4, which is highly tolerant to glyphosate because its EPSPS is less sensitive to inhibition by glyphosate than EPSPS found in plants. The CP4‐EPSPS enzyme has an extremely high inhibition constant, Ki, for glyphosate and low Km for the substrate phosphoenolpyruvate (PEP) (Funke *et al*., 2006; Padgette *et al*., 1995). Several other EPSPS variants from *Escherichia coli* K12 with a reduced affinity for glyphosate were developed through simultaneous Gly96Ala and Ala183Thr substitutions and tested for their efficiency in *Nicotiana tabacum* (Kahrizi *et al*., 2007). Glyphosate‐tolerant corn (Zea mays) event GA21 was developed with a modified *EPSPS* gene from corn driven by a constitutive rice *Actin* promoter with low affinity to glyphosate (Monsanto Safety report, 2002).

Although herbicide‐tolerant transgenic plants are widely cultivated, still there are concerns regarding their adoption in many countries. The herbicide residue in crop plants is a major concern. The accumulation of glyphosate (3.3 mg/kg) and amino methyl phosphonic acid (AMPA) (5.7 mg/kg) in herbicide‐resistant soya bean seeds was reported that it was not found in conventional and organic soya bean batches (Bohn *et al*., 2014). The long‐term glyphosate toxicity studies using Roundup‐tolerant NK603 genetically modified maize material showed liver and kidney toxicity at very low levels (Mesnage *et al*., 2015). From this context, transgenic plants expressing genes that can modify or detoxify glyphosate from plant source have greater significance (Rommens *et al*., 2004; Schouten *et al*., 2006). Transgenic plants of *Arabidopsis*, tobacco and maize have been developed with engineered *Glyphosate N‐acetyltransferase* (*GAT*) gene from *Bacillus licheniformis* acetylate glyphosate (Castle *et al*., 2004; Siehl *et al*., 2005). Similarly, *Glyphosate oxidoreductase* (*GOX*) and mutated *Glycine oxidase* (*GO*) from *Bacillus subtilis* cleave the carbon–nitrogen bond in glyphosate and yield aminomethylphosphonic acid (AMPA), which is less phytotoxic (Pedotti *et al*., 2009; Pollegioni *et al*., 2011; Franz *et al*., 1997). Due to the potential benefits of using bacterial GOX and GO, transgenic plants have been developed by expressing these genes in plants to reduce glyphosate residual effect (Nandula *et al*., 2005). Several *Pseudomonas* sp. strains have been reported to utilize glyphosate and degrade it into glycine and produce CO_2_. These strains use glyphosate as a sole phosphorus source and they play a role in phytoremediation to degrade toxic compounds (Hove‐Jensen *et al*., 2014). The *increased glyphosate‐resistant gene A* (*igrA*) from *Pseudomonas* strain PG2982 detoxifies glyphosate into sarcosine and inorganic phosphate and subsequently formaldehyde and glycine (Fitzgibbon and Braymer, 1990). In soil, the glyphosate‐detoxifying mechanisms by microbes are well studied. However, glyphosate detoxification by plant endogenous mechanisms has not been identified.

In this study, we characterize aldo‐keto reductase (*AKR1*) gene from *Pseudomonas* and rice (*OsAKR1*). The protein docking studies with glyphosate clearly suggest that AKRs can efficiently bind to glyphosate. The *AKR1*‐expressing *E. coli* cells were able to grow in glyphosate‐supplemented media. We also validated the role of AKRs in glyphosate detoxification by virus‐induced gene silencing (VIGS) in rice and *Nicotiana benthamiana* or mutation of *AKR1* in yeast and *Arabidopsis*. Tobacco transgenic plants expressing *AKR1* showed tolerance to glyphosate. By in vitro regeneration of tobacco explants and screening of putative rice transformants, we show that *AKR1* can be used as a potential selectable marker gene against glyphosate. Our data suggest that AKR proteins from plants can be used to develop transgenic plants that can tolerate glyphosate and at the same time reduce residual levels in plants.

## Results

### The *igrA* encodes an aldo‐keto reductase (AKR1) enzyme

The *igrA* (Acc. No. M37389) from *Pseudomonas* sp. strain PG2982 was reported to detoxify glyphosate in *E. coli* (Fitzgibbon and Braymer, 1990). Based on bioinformatic analysis, we determined that *igrA* homologues identified in different organisms belong to NADPH‐dependent aldo‐keto reductase (AKR) superfamily. The *igrA* has 40% homology at amino acid level to several characterized AKRs. The nearest neighbour analysis of characterized *AKR* sequences shows that *igrA* belongs to the AKR family (Figure S1a); hence, igrA gene will be referred as *Pseudomonas AKR1* (*PsAKR1*). Alignment of PsAKR1 amino acid sequence with other AKR sequences using SMS2.0 (sequence manipulation tool) identified highly conserved sequences (Figure S2). PsAKR1 showed 24% homology with OsALR1 (aldose reductase) (NP_001055731.2) and 28% with OsAKR1 proteins (ABF97586.1; Figure S3). The NCBI conserved domain tool (http://www.ncbi.nlm.nih.gov/Structure/cdd/wrpsb.cgi) to predict conserved structure using CDDv3.10‐44354 PSSM database shows aldo‐keto reductase domains (Figure S4), indicating this protein has all the characteristic features of aldo‐keto reductase.

### PsAKR1 and OsAKR1 bind to glyphosate

**Figure 1:**
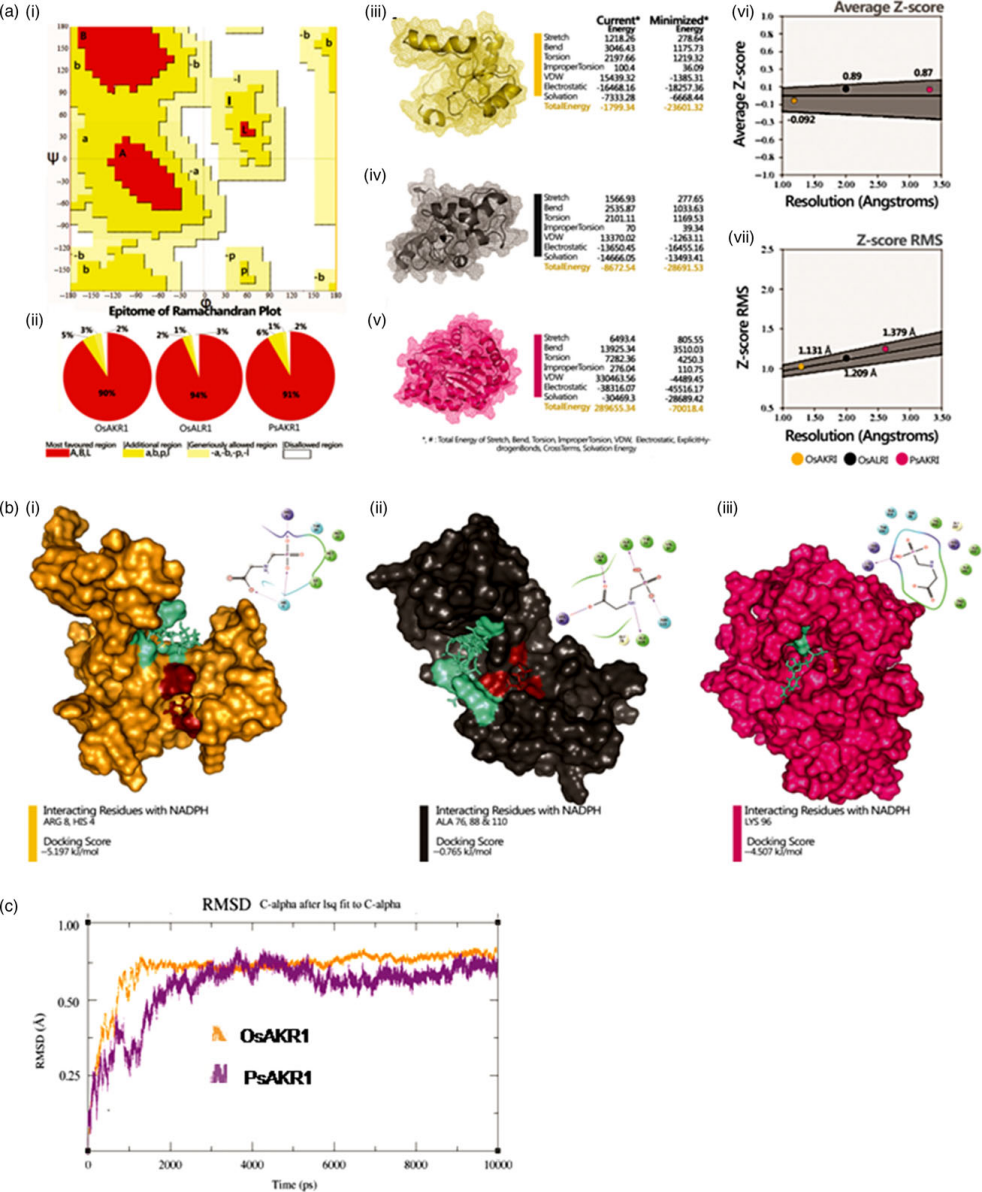
AKR proteins binds to glyphosate efficiently, (a) structure validation, (i) and (ii) epitome of Ramachandran plot for phase I validation of structures by conformational analysis showing classified favourable and nonfavourable regions of modelled proteins’ amino acid plotted against rotamer library (A—right‐handed 3‐10 alpha helix, B—beta sheets, P—polystrand, L—left‐handed alpha helix), (iii) OsAKR1, (iv) OsALR1, (v) PsAKR1—phase II validation based on energy analysis—energy calculation of pre‐ and postminimized structures of modelled proteins by Schrodinger's macromodel module, (vi) average *Z*‐score, (viii) *Z*‐score RMS—phase III validation based on scoring analysis—cumulative scoring and RMS calculations of each modelled structure by protein volume evaluation (PROVE) from SAVES server. (b) Molecular docking of secondary phase of NADPH complexes with OsAKR1, OsALR1 and PsAKR1 with glyphosate, (i) 2D and 3D interactive map of OsAKR1 complex with glyphosate, (ii) OsALR1 with glyphosate, (iii) PsAKR1 with glyphosate. (c) Molecular dynamic simulation of NADPH complexes of OsAKR1 and PsAKR1 with glyphosate for 10 nanoseconds and 10 000 trajectories. AKRs bind to glyphosate which has C=O in its structure. The docking studies confirm that OsAKR1 binds to glyphosate efficiently than OsALR1. (For more details on docking please see Supplementary information I.).

Glyphosate has carbonyl (C=O, keto‐group) group in its structure (Samsel and Seneff, 2013) and this group containing reactive compounds are substrates for AKR enzymes (Sanli *et al*., 2003). To understand whether or not AKRs bind to glyphosate, we developed protein structures by modelling using Schrodinger tool (Figure 1 & supplementary information I) (Schrödinger Release 2015). The modelled proteins were validated based on conformational, energy and score‐based profiles against Ramachandran's conformation library (Sastry *et al*., 2013). From the rotamer library, there are 90%, 91% and 94% of most favourable region in OsAKR1, OsALR1 and PsAKR1 structures, respectively. Further, 5%, 2% and 6% of additional allowed region; 3%, 1% and 1% of generously allowed region; and 2%, 3% and 2% of disallowed region from OsAKR1, OsALR1 and PsAKR1 structures were observed, respectively (Figure 1a‐i‐ii). All the proteins show the presence of eight α‐β sheets, which is a characteristic structural feature of AKRs. The conformational structures were analysed for current energy profile and minimized energy profile. Current and total energy of OsAKR1 were −1799.34 and −2360.32, of OsALR1 were 8672.54 and −28691.53 and of PsAKR1 were 289655.34 and −70018.4, respectively, which corresponds to stretch energy (strE), bend energy (benE), torsion energy (torE), improper torsion energy (impE), vDW energy (vDWE), electrostatic energy (elecE) and solvation energy (solE) (Figure 1a‐iii‐v). The structures of postanalysed modelled proteins were subjected to calculation of the average *Z*‐score and root‐mean‐square deviation (rms) to find cumulative frequencies. Average *Z*‐score of OsAKR1, OsALR1 and PsAKR1 is −0.92, 0.89 and 0.87, respectively. Angstrom and *Z*‐score rms is 1.131 A, 1.2.9 A and 1.379 A, respectively (Figure 1a‐vi‐vii). The validated modelled proteins were first interacted with NADPH because AKR proteins bind to cofactor before binding to substrates. Phase I docking scores for OsAKR1, OsALR1 and PsAKR1 with NADPH are −9.598, −10.081 and −10.892 kJ/mol, respectively. For NADPH molecule, the binding affinity at ARG 8, PHE 7, HIS 4, ASN 49, GLN 70, GLN 2 and VAL 95 with *OsAKR1*. Similar to NAPDH, OsALR1 occupies at ARG 80, ALA 78, SER 43 and PsAKR1 at ASP 257, ALA 269, ALA 291, ARG 195, ARG, 266, TYR 138 amino acids (Figure S4b). Further, these complexes of each protein were subjected to interaction with glyphosate. Phase II docking profile gives −5.197, −0.765 and −4.507 kJ/mol for complex of OsAKR1, OsALR1 and PsAKR1 with glyphosate. The occupancy of glyphosate in each protein was close to NADPH‐interacted regions with ARG 8, HIS 4 from OsAKR1, ALA 76, ALA 88, ALA 110 from OsALR1 and LYS 98 from PsAKR1 structure (Figure 1b). Based on the docking scores, we further evaluated the AKR–NADPH–glyphosate complexes of OsAKR1 and PsAKR1 with glyphosate using molecular dynamic (MD) simulation (Sander Pronk *et al*., 2013). The stable deviation range value for PsAKR1 is 5.7–10 ns and for OsAKR1 is 0.7–10 ns of simulation period. Superpositioned deviation values are 0.74 for PsAKR1 and 0.68 A for OsAKR1, which suggests that PsAKR1 can bind more efficiently to glyphosate (Figure 1c).

### *AKR1*‐expressing bacterial cells and tobacco transgenic plants are tolerant to glyphosate

**Figure 2:**
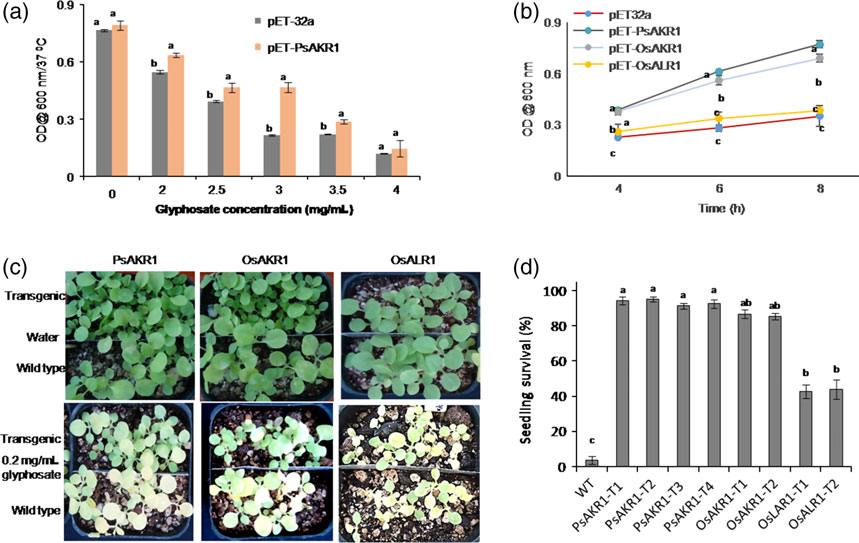
Response of *E. coli* cells and tobacco transgenics expressing *AKR* genes on glyphosate, (a) growth of bacterial cells in media containing different concentrations of glyphosate (n = 5/concentration), (b) comparative growth assessment of *PsAKR1*‐, *OsAKR1*‐ and *OsALR1*‐expressing cells in 3 mg/mL of glyphosate. The error bars represent the standard deviation for three replications. Different letters above the bars indicate significant difference. (c) Response of transgenic and wild‐type seeds on 0.2 mg/mL of glyphosate. Glyphosate was sprayed on 15‐day‐old seedlings of both transgenic and wild‐type seedlings. Photographs were taken after 5 days of spraying. (d) Survival rate of transgenic seedlings and wild‐type seedlings upon glyphosate spraying. Minimum of 25 seeds were used in each replication and three replications were maintained. Experiments were repeated three times. Different letters above the bars indicate a significant difference from two‐way ANOVA at *P* < 0.05 with Tukey's honest significant difference (HSD) means separation test (*a* = 0.05) with wild‐type and transgenic plants and mutant strains. The experiment was repeated for a minimum of three times. Two‐way ANOVA at *P* < 0.05 with Tukey's HSD means separation test (*a* = 0.05) among WT with mutants were conducted to analyse the significance differences in growth.

To validate the relevance of *PsAKR1* in providing resistance against glyphosate in plants, the gene was codon‐optimized to plants and synthesized (Figure S5). This *PsAKR1* was expressed in *E. coli* pET32a expression vector. The *PsAKR1*‐expressing bacteria grew significantly more than the vector control after 8 h of incubation at different concentrations of glyphosate (Figure 2a). Further, to know whether or not plant AKRs also impart tolerance to glyphosate in *E. coli*,* OsAKR1* (Os01 g0847600) and *OsALR1* (Os01 g0847800) genes were expressed in *E. coli*. pET32a‐*OsAKR1* culture showed significantly higher growth than pET32a‐*OsALR1* and pET32a empty vectors at 3 mg/mL of glyphosate (Figure 2b). The pET32a‐*OsALR1* culture could grow very slowly on glyphosate.

To assess the response of *PsAKR1*,* OsAKR1* or *OsALR1* in plants, the gene constructs driven by constitutive promoter ribulose 1, 5‐bis phosphate carboxylase small subunit (*RBCS*) were transformed to tobacco plants. The transformants were efficiently regenerated on glyphosate media (Figure S6a&b). Further, the transgenic seeds expressing *PsAKR1*,* OsAKR1* or *OsALR1* were germinated on glyphosate (Table S1) and expression of the transgene was confirmed in transgenic plants by qRT‐PCR (Figure S6c). The 15‐day‐old *PsAKR1*,* OsAKR1* and *OsALR1* expressing transgenic seedlings were sprayed with 0.2 mg/mL of glyphosate. The *AKR*‐expressing seedlings showed significantly less chlorosis and higher tolerance to glyphosate (Figure 2c). However, *OsALR1* transgenic seedlings showed early chlorosis and most of the seedlings did not survive after glyphosate treatment as similar to wild‐type seedlings (Figure 2d).

### *AKR1*‐silenced rice and *N. benthamiana* and yeast, *Arabidopsis akr* mutant plants are hypersensitive to glyphosate

**Figure 3:**
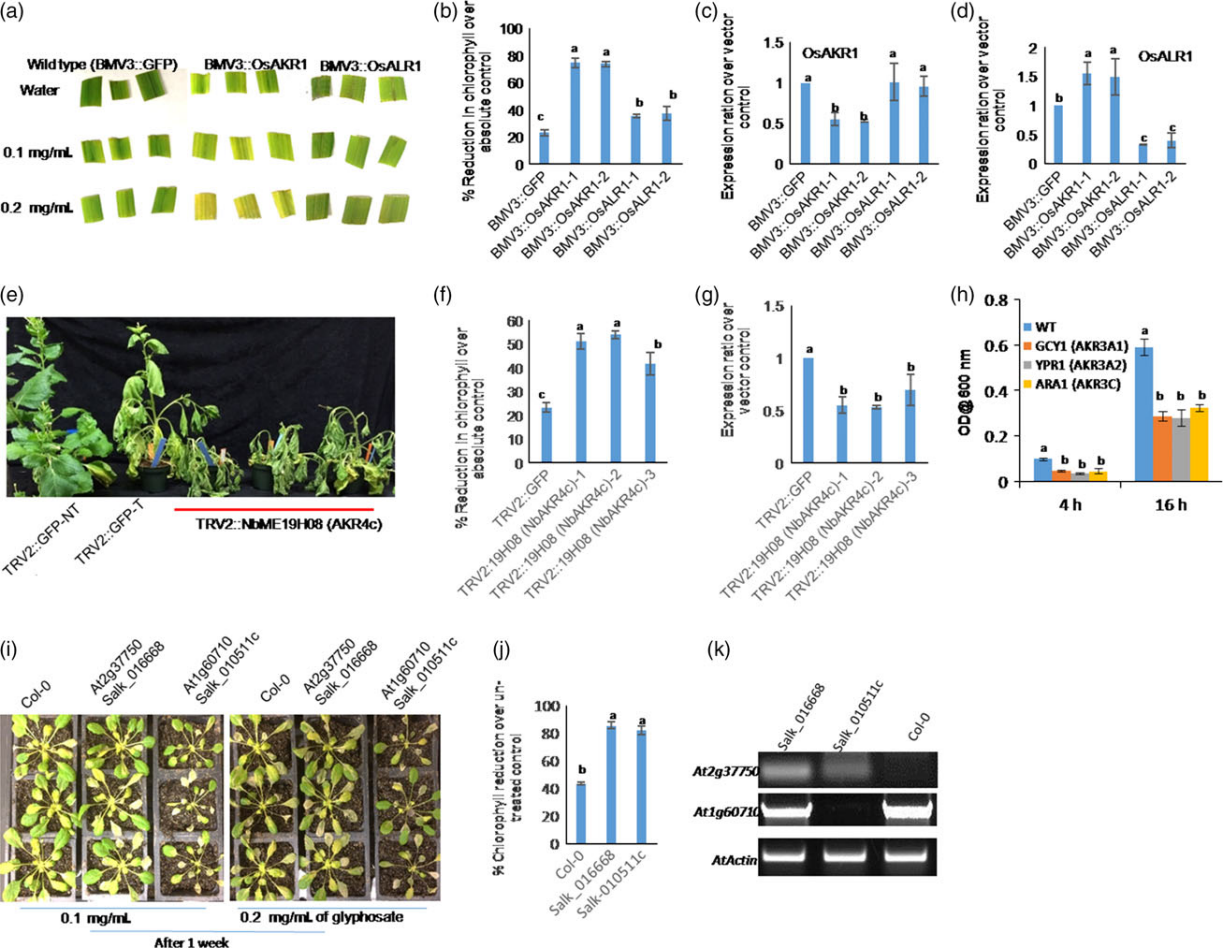
Response of *AKR*‐silenced and mutant yeast strains and plants against glyphosate, (a–d) *OsAKR1*‐ and *OsALR1*‐silenced rice plants by virus‐induced gene silencing (VIGS), (a) phenotypic response of leaf discs from 30‐day‐old silenced plants treated with 0.1 and 0.2 mg/mL of glyphosate and exposed to mild light for 48 h, (b) chlorophyll reduction over untreated leaf discs, (c) and (d) expression of *OsAKR1* and *OsALR1* in silenced plants (n = 50 seedlings per construct used for silencing), (e–g) silencing of *NbAKR* in *N. benthamiana* using TRV‐VIGS, (e) phenotype of *AKR*‐silenced *N. benthamiana* plants sprayed with 0.5 mg/mL of glyphosate after 3 weeks of silencing, (f) chlorophyll reduction over water control, (g) expression analysis of *AKR1* in VIGS plants using qRT‐PCR (n = 25 plants used for silencing). (h) Response of yeast *S. cerevisiae* wild‐type and mutant strains lacking different AKRs on YPD media supplemented with glyphosate, YPR1—NADPH‐dependent AKR, ARA1—arabinose dehydrogenase, GCY1—glyceraldehyde dehydrogenase. Growth response of yeast strains on normal and 1 mg/mL of glyphosate. The mutants and wild‐type strains were grown without any glyphosate up to 3 × 10^−3^ and then diluted to 10^−1^, 10^−2^ and 10^−3^ concentrations and 5 μL from each was inoculated into YPD broth containing 1 mg/mL of glyphosate. The absorbance at 600 nm was recorded after 4 and 16 h after incubation, (i–k) response of *Arabidopsis* mutants, (i) phenotypic differences between Col‐0 and mutants on 0.1 and 0.2 mg/mL of glyphosate, (j) chlorophyll reduction in mutants treated with 0.2 mg/mL of glyphosate over respective control samples (n = minimum of five seedlings per treatment), (k) semiquantitative PCR to test the expression of AKRs. In respective mutants with gene‐specific primers. Data were obtained from three independent experiments with three biological replicates and the error bars represent standard deviation. Different letters above the bars indicate a significant difference from two‐way ANOVA at *P *< 0.05 with Tukey's HSD means separation test (*a* = 0.05) among respective wild‐type, gene‐silenced or mutant plants.

To investigate the function of the rice *AKR* gene in providing tolerance against glyphosate, we independently silenced *OsAKR1* and *OsALR1* genes in rice (IR‐64) using *Brome mosaic virus* (BMV)‐based virus‐induced gene silencing (VIGS; Ding *et al*., 2006). In leaf disc assays with different concentrations of glyphosate, *OsAKR1*‐silenced plants showed hypersensitive phenotype and reduced chlorophyll content compared with wild‐type plants (Figure 3a, b). Further, spraying 0.5 mg/mL of glyphosate to 30‐day‐old seedlings confirms that the *OsAKR1*‐silenced plants were hypersensitive compared with the *OsALR1*‐silenced and wild‐type plants. In the case of *OsAKR1*‐silenced plants, only 10%–15% of seedlings survived. With *OsALR1* silencing, 70%–85% wild‐type plants survived (Figure S7a & b). Even with 50% of gene silencing, *OsAKR1* caused a hypersensitive phenotype (Figure 3c). Silencing level was 70% in *OsALR1*‐silenced plants (Figure 3d), but they were less sensitive to glyphosate.

To test whether *AKR1* can provide tolerance against glyphosate in another plant species, *N. benthamiana*, we silenced *AKR1* homologue (JZ764666.1) using *Tobacco rattle virus*‐based VIGS (Senthil‐Kumar and Mysore, 2014). Spraying 0.5 mg/mL of glyphosate on *NbAKR1*‐silenced plants caused severe wilting compared with nonsilenced control plants (Figure 3e) with 50%–60% reduction in chlorophyll content (Figure 3f) and 5%–10% survival rate. There was a 70% survival rate in wild‐type plants (Figure S7c). The expression of *NbAKR* in silenced plants was reduced by 50% (Figure 3g). In addition, the growth of yeast (*Saccharomyces cerevisiae*) mutant strains of *AKR* family genes such as *AKR3A1* (Acc. No. P14065), *AKR3A2* (Acc. No. NP010656), *AKR3C* (Acc. No. Z36018) and wild type on 0.5 mg/mL of glyphosate‐supplemented yeast extract–peptone–dextrose agar (YPDA) media was assessed. These mutants showed a susceptible phenotype at 1 mg/mL of glyphosate in YPDA media supplemented with glyphosate and all the mutants showed significantly delayed or slow growth rates than the wild‐type strain (Figure 3h). The *Arabidopsis* mutants of *AKR1* homologues, At2g37750 (Salk‐016668) and At1g60710 (Salk‐010511c; Figure S7d & f), were assessed for glyphosate sensitivity by spraying 0.1 and 0.2 mg/mL of glyphosate to four‐week‐old plants. Both the mutants showed highly sensitive phenotype compared with Col‐0 plants at two concentrations tested (Figure 3i). These mutants showed only 10%–20% germination on 0.02 mg/mL of glyphosate‐supplemented media (Figure S7e), whereas 70%–80% of the wild‐type seeds germinated. Glyphosate significantly reduced chlorophyll content in mutants compared with wild‐type Col‐0 plants (Figure 3j, k) and 10%–20% of plants survived 1 week after treatment (Figure S7f). These data clearly suggest that AKRs are involved in imparting glyphosate tolerance in plants.

### *AKR1*‐overexpressing tobacco leaf explants regenerate on glyphosate

**Figure 4:**
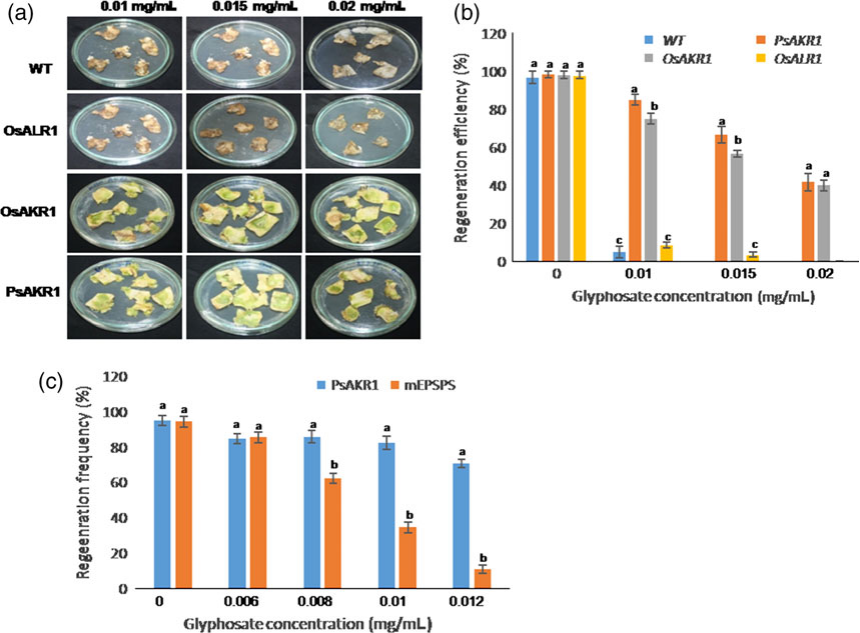
Regeneration efficiency of tobacco plants expressing AKRs on glyphosate selection media, (a) response of tobacco leaf explants expressing *OsAKR1*,* OsALR1*,* PsAKR1* on regeneration media supplemented with different concentrations of glyphosate, (b) the frequency of regeneration was estimated for *PsAKR1*‐, *OsAKR1*‐ and *OsALR1*‐expressing explants on different concentrations of glyphosate on ½ MS media. Minimum of 25(n) explants were maintained for each transformation. (c) Regeneration efficiency of *PsAKR1* and mutant *EPSPS*‐expressing explants. The leaf discs from transgenic tobacco expressing *PsAKR1* and *mEPSPS* plants were regenerated on different concentrations of glyphosate media. Minimum of 25 leaf discs from each transgenic plants were used to assess in three different experiments. Different letters above the bars indicate a significant difference from two‐way ANOVA at *P* < 0.05 with Tukey's HSD means separation test (*a* = 0.05) with wild‐type and transgenic plants.

To test whether the *AKR* genes when transformed to plants show regeneration on glyphosate‐supplemented Murashige and Skoog (MS) media and can be used as a selectable marker, the AKR1‐ and ALR1‐expressing tobacco leaf explants were assessed for regeneration. The *PsAKR1*‐expressing explants were used to standardize the lethal dose and with 0.012 mg/mL of glyphosate, more than 50% of the explants survived (Table S2). Further, to compare the regeneration of *PsAKR1*‐, *OsAKR1*‐ or *OsALR1*‐expressing tobacco leaf explants, 0.015 and 0.02 mg/mL of glyphosate were used. Improved regeneration and higher greening of the explants were observed in *PsAKR1*‐ or *OsAKR1*‐expressing explants, whereas explants from wild‐type and *OsALR1*‐expressing plants showed complete browning or death on all concentrations of glyphosate tested (Figure 4a). The frequency of regeneration was significantly higher in *PsAKR1*‐ or *OsAKR1*‐expressing plants than in *OsALR1*‐expressing and wild‐type explants (Figure 4b). Further, the regeneration efficiency of tobacco explants was compared between a 2 × 35*S* promoter driving modified *EPSPS* gene construct and a *PsAKR1* construct. *PsAKR1*‐expressing explants were able to regenerate even at 0.012 mg/mL of glyphosate, whereas the regeneration efficiency of *mEPSPS*‐expressing explants was significantly reduced at concentrations of 0.008 mg/mL glyphosate or more (Figure S8 & Figure 4c). Further, to test whether the PsAKR1 can be used as a selectable marker in other plant systems, rice transgenics expressing *PsAKR1* were developed. The *PsAKR1*‐expressing rice transgenic seedlings were efficiently screened on glyphosate, and subsequently, the survived seedlings were also exposed to leaf swabbing assays (Figure S9). The rice transformants showed tolerance to glyphosate. The regeneration efficiency clearly demonstrates that *OsAKR1* can also function as similar to *PsAKR1* to detoxify glyphosate and it can be used as a selectable marker gene against glyphosate to identify stable transformants.

### Shikimic acid pathway was unaffected by glyphosate in *PsAKR1*‐expressing transgenic tobacco plants

**Figure 5:**
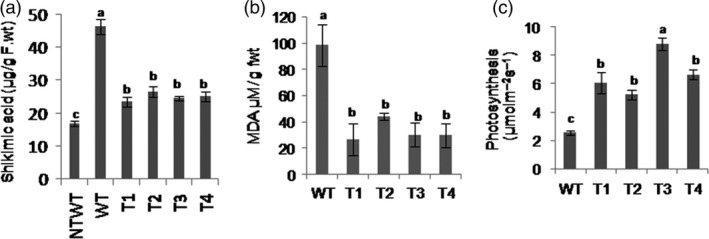
Effect of glyphosate on *PsAKR1*‐expressing transgenics, (a) shikimic acid levels in glyphosate‐treated plants. Shikimic acid was measured from the leaves collected 5 days after the plants were sprayed with 1 mg/mL of glyphosate, (b) MDA levels in glyphosate‐treated plants, and (c) photosynthetic rate. Effect of glyphosate on photosynthetic machinery was studied 4 days after using LICOR‐IRGA machine. Six‐week‐old (50‐day‐old) plants in pots were maintained in glasshouse conditions. Glyphosate (1 mg/mL) was sprayed to both wild‐type and transgenic tobacco plants (n = 25). Photographs were taken after a week. Different letters above the bars indicate a significant difference from two‐way ANOVA at *P* < 0.05 with Tukey's HSD means separation test (*a* = 0.05) with wild‐type and *PsAKR1*‐expressing transgenic plants.

Glyphosate inhibits the activity of EPSPS that catalyses the reaction of PEP and 3‐phosphoshikimate into 5‐enolpyruvylshikimate‐3‐phosphate, which results in accumulation of shikimic acid. If AKRs detoxify glyphosate, the EPSPS enzyme activity will be unaffected and shikimic acid levels will be less in transgenic plants. The 50‐day‐old *PsAKR1* transgenic and wild‐type tobacco plants were sprayed with 1 mg/mL of glyphosate. Wild‐type plants showed wilting phenotype within 5 days and also leaf burning and stem rotting, whereas *PsAKR1* transgenic plants did not show any of these symptoms even 15 days after glyphosate spraying. The transgenic plants showed significantly less accumulation of shikimic acid than wild‐type plants, indicating that EPSPS activity in transgenic plants was not affected by glyphosate (Figure 5a). The glyphosate‐induced reactive oxygen species (ROS) leading to the formation of the lipid peroxidation end product such as malondialdehyde (MDA) in transgenic plants was significantly less than the wild‐type plants (Figure 5b). The transgenic plants showed significantly higher photosynthetic rate than wild‐type plants (Figure 5c).

### AKR proteins degrade glyphosate and rescued cucumber seedlings from glyphosate inhibition

**Figure 6:**
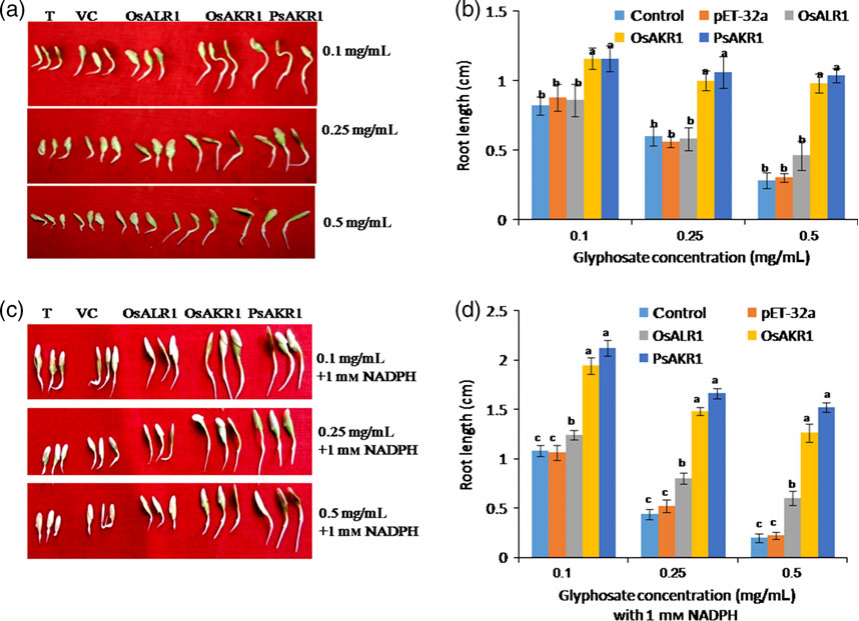
AKRs degrade glyphosate to rescue cucumber seedlings from inhibition, (a) growth of cucumber seedlings on different concentration of glyphosate, T—without any crude protein from bacteria, VT—vector control protein, (b) root growth of cucumber seedlings incubated with crude protein mix of AKRs at different concentrations of glyphosate treatments, (c) addition of NADPH (1 mm) with crude protein mix of AKRs and different concentrations of glyphosate improved the recovery of seedlings, (d) root growth of cucumber seedlings with glyphosate, AKRs and 1 mm NADPH mix. The OsALR1 protein mix added seedling did not recover the levels of PsAKR1 and OsAKR1, confirming AKRs specifically degrade glyphosate (n = 10). Experiments were repeated three times. Different letters above the bars indicate a significant difference from two‐way ANOVA at *P* < 0.05 with Tukey's HSD means separation test (*a* = 0.05) among empty vector with AKRs.

The ability of AKR and ALR1 proteins to degrade glyphosate was assessed in the crude protein extracts from *E. coli* strains expressing these genes. The crude protein extract was incubated with different concentrations of glyphosate (0.1, 0.25 and 0.5 mg/mL) for 3 h. Subsequently, the cucumber seedling growth was assessed using the assay media as a indirect measure of residual levels of glyphosate in the media. The seedlings treated with PsAKR1 and OsAKR1 protein extracts showed improved growth on glyphosate even at 0.5 mg/mL compared with OsALR1 and vector control (Figure 6a). The root growth of cucumber seedlings in PsAKR1 and OsAKR1 extracts was also significantly higher than that of OsALR1 and vector control extracts (Figure 6b). This study clearly demonstrates that ectopically supplied AKRs but not ALR can detoxify glyphosate and rescue cucumber seedlings from glyphosate inhibition. As AKRs are NADPH dependent and to know whether or not the supply of cofactor enhances the activity of AKRs, 1 mm NADPH was added to the protein extracts with glyphosate and incubated for 1.5 h and cucumber seedling growth was assessed using this assay media. The germination and growth of seedlings were significantly higher in PsAKR1 and OsAKR1 proteins with NADPH than in OsALR1 and vector control extracts (Figure 6c). The NADPH addition also enhanced the rate of root growth in medium containing PsAKR1 and OsAKR1 proteins compared with OsALR1 and vector control (Figure 6d). Interestingly, we observed weak activity of OsALR1 against glyphosate. The similar response was observed with crude protein extracts from tobacco transgenic plants expressing AKRs and OsALR1. Results indicate that higher root growth in cucumber seedling treated with crude leaf protein extracts of PsAKR1 and OsAKR1 was observed. However, the cucumber seedling growth was highly inhibited in the OsALR1 and wild‐type leaf extracts, indicating less degradation of glyphosate (Figure S10). These data demonstrate that ectopically applied AKR proteins can rescue cucumber seedling growth inhibited by glyphosate.

## Discussion

In recent years, the glyphosate residual levels in food water as well as human exposures have raised global concerns. In humans, the glyphosate residue causes endocrine disruption by increasing oxidative stress (Bohn *et al.,* 2014; Mesnage *et al*., 2015) and also causes gluten intolerance by impairing activity of many cytochrome P450 enzymes (Samsel and Seneff, 2013). Therefore, it is highly important to develop crop plants with no glyphosate residual toxicity using detoxifying mechanisms. The glyphosate‐detoxifying genes from microorganisms that utilize glyphosate as nitrogen and phosphorous sources have been identified and characterized (Hove‐Jensen *et al*., 2014; Fitzgibbon and Braymer, 1990). We characterized a previously identified *igrA* gene from *Pseudomonas* species strain PG2982. Dalrymple *et al*. (1992) reported that the open reading frame of the *igrA* locus has similarity to *AKR* genes. However, *igrA* has been excluded from the nomenclature of the AKR superfamily due to lack of functional characterization (http://www.med.upenn.edu/akr/potential.shtml). This prompted us to characterize the function of putative *AKR1* encoded by *igrA* and its plant orthologs. AKRs represent oxidoreductase superfamily with seven families at 40% amino acid homology. AKRs are found in both eukaryotes and prokaryotes and are known to metabolize a wide range of substrates including aliphatic aldehydes, monosaccharides, steroids, prostaglandins and xenobiotics (Jez and Penning, 2001). The AKR family of enzymes recognizes a variety of carbonyl substrates. However, molecular basis for the differences in substrate recognition among family members is not known (Sanli *et al*., 2003; Sanli and Blaber, 2001). The protein structure of AKRs from *Pseudomonas* and rice contains eight (α‐β) barrel‐shaped motifs, which is consistent with characterized AKR proteins from barley (*Hordium vulgare*), wheat (*Triticum aestivum*) and rice (*Oryza sativa*) (Simpson *et al*., 2009). The most important feature of these enzymes is binding to NADPH cofactor in an extended conformation via an induced‐fit mechanism (Sanli *et al*., 2003; Sanli and Blaber, 2001). Upon examining the modelled protein–NADPH–glyphosate complexes, we discovered that OsAKR1 and *PsAKR1* possess higher binding affinity by juxtapositioning docking profile with MD run of 10‐ns intervals. The OsALR1 binds to glyphosate less efficiently than AKRs. The docking profile and MD run suggest PsAKR1 and OsAKR1 can bind and catalyse glyphosate more efficiently than OsALR1. This suggests carbonyl group in glyphosate determines the specific binding and catalysis by keto reductases. The rescue of cucumber seedlings with the addition of NADPH and glyphosate (Figure 6) provides additional evidence that exogenous supply of cofactor improves AKRs function for detoxification. However, more detailed biochemical studies are needed to confirm this possibility.

The bioefficacy studies using different systems show that AKRs are involved in detoxification of glyphosate. The studies on yeast mutants for *AKR3A1*,* AKR3A2* and *AKR3C* genes showed a hypersensitive phenotype to glyphosate. Similarly, the silencing of *OsAKR1* in rice and *NbAKR1* in *N. benthamiana* and characterization of *Arabidopsis akr1* mutants also showed a hypersensitive phenotype to glyphosate. The *E. coli* expressing *AKRs* and tobacco transgenic expressing these genes showed improved tolerance against glyphosate. However, the response was only observed in *AKR1*‐expressing system and not in *ALR1*‐expressing transgenics. This suggests that even though ALR1 belongs to AKR family, it did not provide tolerance against glyphosate. The protein docking studies of *ALR1* showed less binding affinity to glyphosate. In the *PsAKR1*‐expressing tobacco transgenic plants after glyphosate treatment, low shikimic acid contents suggest that the plant EPSPS enzyme was not inhibited by glyphosate and hence the aromatic amino acid biosynthesis was not affected. This suggests AKRs detoxify glyphosate before reaching its target. Glyphosate has been showed to represses the photosynthetic activity by inhibiting CO_2_ assimilation and reduce carbon reduction cycle (Geiger *et al*., 1986). The transcriptomic assays identified repression of photosynthetic genes, chlorophyll biosynthesis and Calvin cycle enzymes in *Festuca* species with increased flux of shikimic acid and reduced *EPSPS* expression after 5 days of glyphosate treatment (Cebeci and Budak, 2009). The *AKR1*‐expressing plants also showed higher photosynthetic rates. The CO_2_ assimilation was not affected in transgenic plants and resulted in higher photosynthetic rates with higher biomass, suggesting that AKRs can potentially decrease the glyphosate levels and thus maintain the plant photosynthetic machinery. The transgenic plants also showed less accumulation of lipid peroxidation end products such as MDA. This could be due to detoxification of glyphosate or scavenging reactive carbonyls induced by glyphosate. The *E. coli* expressing *OsAKR1* showed abiotic stress tolerance by detoxifying carbonyl containing compound methylglyoxal (Turóczy *et al*., 2011). The *Medicago sativa ALR* belonging to the *AKR* family in tobacco plants also showed tolerance to hydrogen peroxide, paraquat, salt or dehydration stresses by reducing reactive carbonyl compounds (Bartels, 2001). However, the expression of *OsALR1* in *E. coli* or tobacco did not show tolerance to glyphosate, suggesting that the observed tolerance of *AKR1*‐expressing plants is mainly through detoxification of glyphosate. The glyphosate detoxification is also evident by residual toxicity assessment by cucumber seedling assay. The residual levels of glyphosate were significantly less in AKR1‐treated proteins evident by higher root and shoot growth of cucumber seedlings. AKR proteins are NADPH dependent and exogenous supply of cofactor further enhances the degradation of glyphosate and rescued the cucumber seedlings by glyphosate‐induced inhibition. The AKR1 proteins detoxify glyphosate more efficiently than the OsALR1 protein. This also supports docking studies with ALR1 showing less binding affinity towards glyphosate. These experimental evidences clearly demonstrate that overexpression of AKRs detoxifies glyphosate with less residual effect on plant growth.

The tobacco regeneration efficiency of the explants of *PsAKR1*,* OsAKR1*‐expressing transgenics and segregation analysis on glyphosate selection media clearly demonstrates that AKRs improves glyphosate tolerance. The regeneration of *OsAKR1* explants on glyphosate and rice transgenic screening assay to identify transformants clearly demonstrates that AKRs can be used as a potential selectable marker gene against glyphosate. This can serve as an alternative antibiotic resistance marker gene like *basta* that is effectively used as selectable marker against phosphinothricin herbicide (Didier Breyer *et al*., 2014). Overall, the protein docking, *E. coli* cell growth assay, cucumber seedling assay and tobacco regeneration assay suggest that PsAKR1 and OsAKR1 can bind to glyphosate and detoxify efficiently than ALR1. The advantage of *AKR1* is that it can detoxify glyphosate with less residual toxicity and can be used to develop herbicide‐resistant transgenic crops. The widely adopted glyphosate‐tolerant crops express insensitive form of *EPSPS*. The coexpression of *AKRs* and *EPSPS* may enhance tolerance as well as reduce the residual effect of glyphosate. This approach can even minimize the possibility of the development of glyphosate‐resistant weeds. These detoxification enzymes could be a potential target to engineer future crop improvement programmes.

## Experimental procedures

### Plant transformation and regeneration

*Nicotiana tabacum* variety KST‐19 was grown in glasshouse conditions. The young leaf was surface‐sterilized with 0.1% Bavistin and 0.1% HgCl_2_ for 1 min and followed by sterile distilled water wash three times. The leaf was placed on the MS medium supplemented with glyphosate. *Agrobacterium* strain *EHA105* containing *AKR* constructs was transformed to tobacco explants (for vector construction and constructs used in the study, please see Supplementary Information I, Table S3). For selection of *PsAKR1* and *OsAKR1* transformants, glyphosate was used as selectable agent, and for *OsALR1*, kanamycin (100 μg/mL) was used as selectable agent. The regeneration efficiency was calculated based on number of explants survived on glyphosate. The rice transgenic overexpressing *PsAKR1* was developed using an in planta transformation method (Babitha, 2013).

### Virus‐induced gene silencing (VIGS)

BMV‐VIGS: *Agrobacterium tumefaciens* GV3101 containing either BMV‐RNA1, RNA2 and BMV‐RNA3 (Ding *et al*., 2006) with *OsAKR1*, BMV‐RNA3 with *OsALR1* (Table S4) and BMV‐RNA3‐GFP fragment were grown overnight at 28 °C in Luria‐Bertani (LB) medium with 25 μg/mL rifampicin and 100 μg/mL kanamycin (both from Sigma. St. Louis, MO). Cells were harvested and induced in 10% Mannitol, 30 mm MES, pH 5.5 and 200 nm/mL acetosyringone and incubated for 4 h with slow shaking. Cells were harvested and resuspended in infiltration medium (10 mm MES, pH 5.5), the optical density (OD) at 600 nm was adjusted to 0.8. The 7‐day‐old rice‐IR‐64 seedlings were maintained in high humidity, and seedlings were vacuum‐infiltrated using 50–70 lbs for 3 min. The infiltrated seedlings were incubated under high humidity for 48 h and maintained in the glasshouse at 25 °C day and 21 °C night temperatures with 16‐h photoperiod with a light intensity of approximately 140 μmol photons m^−2^ s^−1^. For silencing of *NbME19H08* (*AKR*) gene fragment (Table S4) in *N. benthamiana*, TRV2‐VIGS system was used (Senthil‐Kumar and Mysore, 2014). (For the primers used in this study, please see Table S5.)

### Bioinformatics analysis

To identify the homologous genes, NCBI BLAST tool was used and sequence homology was determined using EMBL multiple ClustalW and SMS2.0 (sequence manipulation tool) tools (Stothard, 2000) and phylogenetic analysis was carried out using MEGA 6. Details for modelling of OsAKR1, PsAKR1 and OsALR1 structure can be found in online materials and methods.

### Cloning and expression of genes in *E. coli*

To assess the efficiency of *PsAKR1*,* OsAKR1* and *OsALR1* genes against glyphosate, the bacterial expression system pET32a was used to express the proteins. The pET32a‐expressing *E. coli* plasmids were mobilized to BL21 expression host. The cultures were grown for 4 h, and 1 μm IPTG was added and subsequently different concentrations of glyphosate was added and incubated at 37 °C for 8 h. The absorbance at 600 nm was monitored at different time intervals. The total proteins were dissolved with lysis buffer and quantified using Bradford's method (Bradford, 1976). This protein mixture was used for cucumber seedling assay.

### Yeast mutant screening

To assess the relevance of yeast *AKR* mutants, we obtained the *S. cerevisiae* mutant strains *GCY1*—glyceraldehyde dehydrogenase (AKR3A1‐Acc. No. P14065), *YPR1*—NADPH‐dependent AKR (AKR3A2‐Acc. No. NP010656) and *ARA1*—arabinose dehydrogenase (AKR3C‐Acc. No. Z36018) and grow them on glyphosate (0.5 mg/mL) containing yeast–potato–dextrose (YPD) medium. The mutants and wild‐type strains were grown without glyphosate up to 5 × 10^−3^ and then diluted to 10^−1^, 10^−2^ and 10^−3^ concentrations and 5 μL from each was inoculated in to YPD broth containing 1 mg/mL of glyphosate. The absorbance at 600 nm was recorded after 4 and 16 h after incubation.

### Glyphosate spraying experiments

The leaf discs from 30‐day‐old rice *OsAKR1*,* OsALR1*‐silenced and wild‐type plants were treated with 0.1, 0.2 and 0.500 mg/mL of glyphosate and exposed to mild light for 48 h, and photographs were taken and chlorophyll content and survival rate were recorded. Each time, a minimum of 25 seedlings were silenced and assessed for glyphosate resistance. Similarly, four‐week‐old *N. benthamiana* plants were silenced with *NbME19H08* gene. Both silenced and wild‐type plants were sprayed with 0.5 mg/mL of glyphosate and chlorophyll content and survival rate were recorded.

The *Arabidopsis* Salk mutants, Salk_016668 and Salk_010511c, were obtained from Arabidopsis biological resource centre (http://abrc.osu.edu) (Alonso *et al*., 2003). The mutants were screened on 0.02 mg/mL of glyphosate‐supplemented ½ MS media and survival rate was recorded after 1 week. A minimum of 25 seeds were used to germinate, and experiments were repeated three times. The four‐week‐old seedlings were sprayed with 0.1 and 0.2 mg/mL of glyphosate, and chlorophyll content was measured after 1 week and survival rate was measured after 10 days.

#### Shikimic acid quantification

The shikimic acid content was estimated in the glyphosate‐treated and nontreated plants. The leaves were dried at 65 °C for 72 h and ground well to a fine powder; 100 mg of tissue was treated with 4 mL of 0.01M H_2_SO_4_ and incubated on a rotary shaker water bath for 2 h at 50 °C. The samples were cooled down to room temperature and 1 mL of 0.4 M NaHCO_3_ was added and spin at 36 223 *g* for 20 min at 4 °C. The supernatant was filtered through 0.2‐μm filters and 20 μL sample was loaded to HPLC cuvette to estimate the level of shikimic acid (Zelaya *et al*., 2011). For standard preparation, pure shikimic acid was used (Sigma St. Louis, MO).

#### Cucumber seedling assay

To assess the tolerance efficiency of AKRs and OsALR1 to glyphosate, 3 μg/mL of crude protein from PsAKR1‐, OsAKR1‐ and OsALR1‐expressing recombinant bacterial extract was mixed in 3 mL of water with 0.1, 0.25 and 0.5 mg/mL of glyphosate. Subsequently, this mixture was treated to pregerminated cucumber seedlings. The growth of cucumber seedlings was measured by quantifying root growth. In another set of experiment, 1 mm NADPH cofactor was mixed with glyphosate and crude protein and seeds were germinated on blotting paper, and the experiment was repeated for at least three times. To test whether plants expressing AKR proteins also degrade glyphosate or not, total protein from four‐week‐old plants were extracted using PBS buffer with 1 mm PMSF. And 10 μg of crude protein was mixed with 0.5 mg/mL of glyphosate and incubated at room temperature for 1 h. Subsequently, the pregerminated cucumber seedlings were treated with this mixture and assessed for growth inhibition and root growth.

### Statistical analysis

The data obtained in different experimental results was analysed using two‐way analysis of variance (ANOVA) as per the procedure given by Fisher (1960). Data points with different lowercase letters indicate significant differences (*P* < 0.05) between transgenic lines and wild type as determined by Tukey's HSD test.

## Accession codes

PsAKR1/igrA‐Acc. No. M37389; OsAKR1 ‐ Os01 g0847600; OsALR1‐AK100718; GCY1‐AKR3A1‐Acc. No. P14065; YPR1‐AKR3A2‐Acc. No. NP010656; ARA1‐AKR3C‐Acc. No. Z36018; At2g37750; At1g60710.

## Conflict interest

V.S. R., K.C.B. and M.U.K. are authors of a patent filed in the Indian patent agency on selectable marker system (File No. 154/CHE/2012). However, the authors declare no conflict of interest in the published data.

## Author contributions

VSR, MUK and KSM conceived the project. VSR, ARV and BKC conducted majority of work. ME did protein docking experiments, HR and KG involved in development of transgenic. VSR, BKC, CS, KSM and MUK wrote the manuscript.

## Supporting information


**Figure S1** Phylogenetic analysis of PsAKR1 (igrA) with AKRs from other species.
**Figure S2** Homology of PsAKR1 with other characterized proteins.
**Figure S3** Homology of PsAKR1 with OsAKR1 and OsALR1.
**Figure S4** Conserved domains prediction on PsAKR1 protein and molecular docking of AKR proteins with cofactor NADPH.
**Figure S5** Codon optimization of *PsAKR1* gene to plants.
**Figure S6** Regeneration of tobacco explants transformed with *AKR* and *OsALR1* gene constructs.
**Figure S7** Response of Os*AKR1*‐ and Os*ALR1*‐silenced rice, *N. benthamiana* and Arabidopsis plants against glyphosate.
**Figure S8** Regeneration efficiency of *PsAKR1* and *mEPSPS* expressing transgenic plants.
**Figure S9** Response of rice transgenic plants expressing *PsAKR1* on glyphosate.
**Figure S10** Degradation of glyphosate by AKR proteins from plant.

**Table S1** Screening and segregation analysis of the transgenic tobacco expressing *PsAKR1*,* OsAKR1* and *OsALR1* plants in T1 generation.
**Table S2** Lethal dose (LD) determination of tobacco explants.
**Table S3** Gene constructs used in this study.
**Table S4** Gene fragments used to silence *N. benthamiana AKR* and *OsAKR1* and *OsALR1* genes in rice.
**Table S5** Primers used in this study.

**Appendix S1** Materials and methods related to vector construction and Modeling of OsAKR1, PsAKR1 and OsALR1 structure.
