# Supplementary material for: Aldo‐keto reductase enzymes detoxify glyphosate and improve herbicide resistance in plants
Source: Plant Biotechnol J. 2017 May 11;15(7):794–804. doi: 10.1111/pbi.12632 (PMC5466437; doi:10.1111/pbi.12632)
Supplement: Supplementary file 1 — Figure S1 Phylogenetic analysis of PsAKR1 (igrA) with AKRs from other species. Figure S2 Homology of PsAKR1 with other characterized proteins. Figure S3 Homology of PsAKR1 with OsAKR1 and OsALR1. Figure S4 Conserved domains prediction on PsAKR1 protein and molecular docking of AKR proteins with cofactor NADPH. Figure S5 Codon optimization of PsAKR1 gene to plants. Figure S6 Regeneration of tobacco explants transformed with AKR and OsALR1 gene constructs. Figure S7 Response of OsAKR1‐ and OsALR1‐silenced rice, N. benthamiana and Arabidopsis plants against glyphosate. Figure S8 Regeneration efficiency of PsAKR1 and mEPSPS expressing transgenic plants. Figure S9 Response of rice transgenic plants expressing PsAKR1 on glyphosate. Figure S10 Degradation of glyphosate by AKR proteins from plant. [file PBI-15-794-s002.pdf]

a)

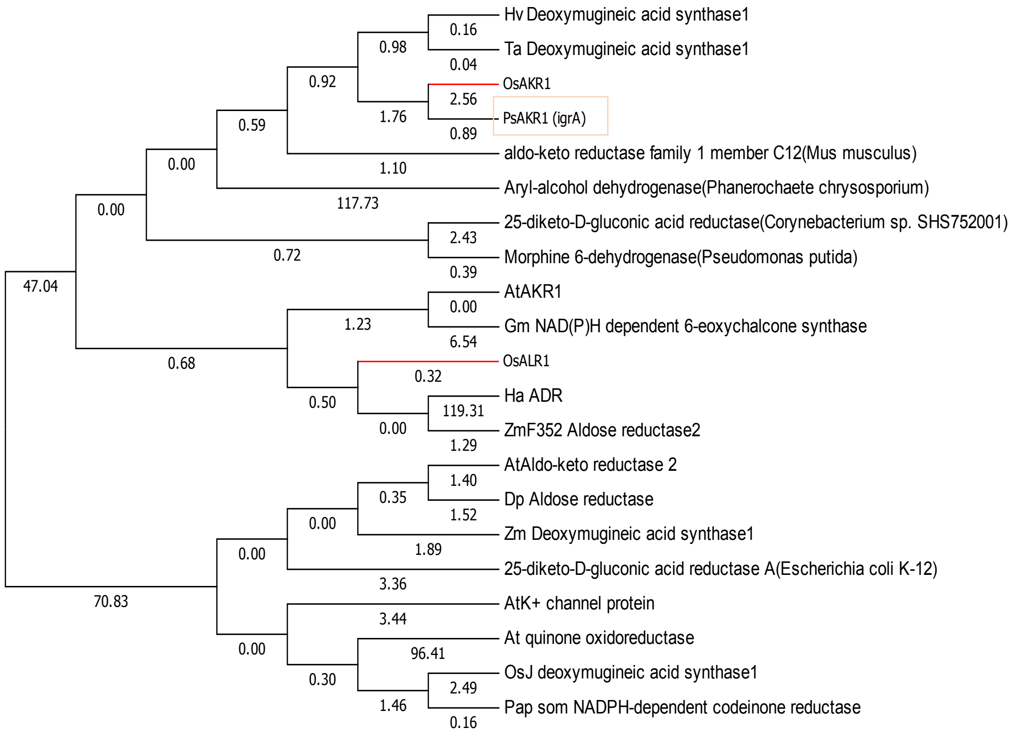
Hv Deoxymugineic acid synthase1
Ta Deoxymugineic acid synthase1
OsAKR1
PsAKR1 (igrA)
aldo-keto reductase family 1 member C12(Mus musculus)
Aryl-alcohol dehydrogenase(Phanerochaete chrysosporium)
25-diketo-D-gluconic acid reductase(Corynebacterium sp. SHS752001)
Morphine 6-dehydrogenase(Pseudomonas putida)
AtAKR1
Gm NAD(P)H dependent 6-eoxychalcone synthase
OsALR1
Ha ADR
ZmF352 Aldose reductase2
AtAldo-keto reductase 2
Dp Aldose reductase
Zm Deoxymugineic acid synthase1
25-diketo-D-gluconic acid reductase A(Escherichia coli K-12)
AtK+ channel protein
At quinone oxidoreductase
OsJ deoxymugineic acid synthase1
Pap som NADPH-dependent codeinone reductase
0.16
0.04
0.98
0.92
2.56
1.76
0.89
0.59
1.10
0.00
117.73
0.00
2.43
0.72
0.39
47.04
0.00
1.23
6.54
0.68
0.32
0.50
119.31
0.00
1.29
1.40
0.35
1.52
0.00
1.89
0.00
3.36
70.83
3.44
0.00
96.41
2.49
0.30
1.46
0.16

b)

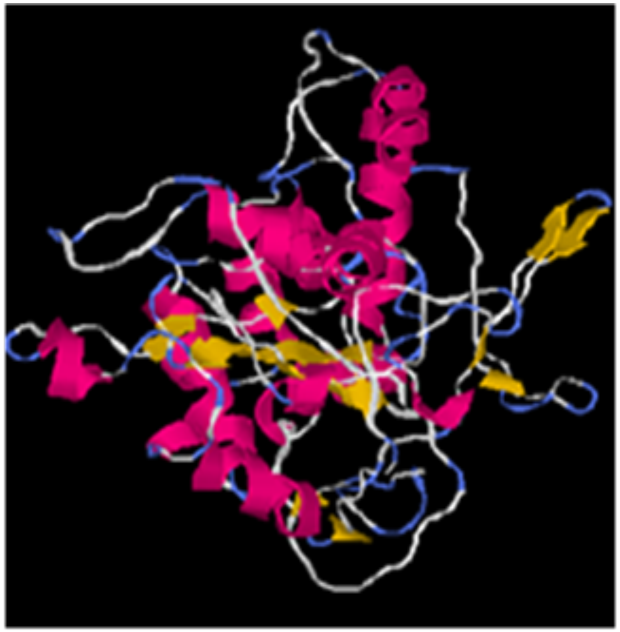

Figure S1.

Figure S1. Phylogenetic analysis of PsAKR1(igrA) with AKRs from other species. a) Molecular phylogenetic analysis by Maximum Likelihood method, The evolutionary history was inferred by using the Maximum Likelihood method based on the JTT matrix-based model (Jones et al 1992) . The tree with the highest log likelihood (-8348.1649) is shown. Initial tree(s) for the heuristic search were obtained automatically by applying Neighbor-Join and BioNJ algorithms to a matrix of pairwise distances estimated using a JTT model, and then selecting the topology with superior log likelihood value. Evolutionary analyses were conducted in MEGA6 [Tamura K., 2013]. The *AKRs* from barley (*Hordium vulgare*), wheat (*Triticum aestivum*) and rice (*Oryza sativa*) showed very close homology and other species also had considerable homology with boot strap value of 0.1 and average branch length of 0.5-1. The *PsAKR1* shows 77% homology with *Voltage-gated potassium channel subunit beta-2-like* gene (XM_005974894). The translated BLAST (protein) analysis of *PsAKR1* showed 95% identity with *Ochrmobactrum* AKR (YP_001369824.1), 66% with *Caulobacter* (WP_007662458.1), 64% with *Pseudomonas* sp. *CFII68* (WP_018608812.1), 28% with *Arabidopsis thaliana* NAD(P)-linked oxidoreductase superfamily protein) (NP_176268.1), 30% with AKR like protein (BAB09734.1), 35% with Auxin induced protein belonging to AKR family (AAB71960.1), 37% with Rice probable voltage gated potassium channel (NP_001048521.1), 30% with probable OsAKR (NP_001052494.1), 31% with OsAKR like protein (NP_001058874.1) and 50% with rice auxin-induced protein PCNT115 (ABG66080.1). b) 3D structure of PsAKR1 using I –TASSER bioinformatics program presence of 8 α(yellow), β (pink) sheeted motifs. The presence of this motifs indicate that, the protein belongs to the AKR family of enzymes.

```
At_Aldo-keto_reducta --------MADEIGFFQLNTGAKIPSVGLGTWQAAPG---VVGDAVAAAVKIGYQHIDCASRYGN---EIEIGKVLK-KLFDDGVVK-REKLFITSKIWL 84
At_Aldo-keto_reducta --------MAEEIRFFELNTGAKIPSVGLGTWQADPG---LVGNAVDAAVKIGYRHIDCAQIYGN---EKEIGLVLK-KLFDGGVVK-REEMFITSKLWC 84
Dp_Aldose_reductase  --------MAEEIRFFELNTGAKIPSVGLGTWQSSPG---DAAQAVEVAIKCGYRHIDGARLYEN---EKEIGVVLK-KLFDDGVVK-REDLFITSKLWS 84
Zm_F352_Aldose_reduc -----------MARHFVLNTGAKIPSVGLGTWQSDPG---VVGNAVYAAVKAGYRHIDCARVYGN---EKEIGLALK-KLFEEGVVK-REDLFITSKLWN 81
Ha_AR__ADR           MASAKATMGQGEQDHFVLKSGHAMPAVGLGTWRAGSD---TAHSVRTAITEAGYRHVDTAAEYGV---EKEVGKGLK-AAMEAGID--RKDLFVTSKIWC 91
Hv_Deoxymugineic_aci -------------MGAGDRTVAGMPRIGMGTAVQGPK-PDPIRRAVLRAIEIGYRHFDTAAHYET---EAPIGEAAA-EAVRSGAVASRDDLFITSKLWC 82
Ta_Deoxymugineic_aci -------------MGAGDRTAAGMPRIGMGTAVQGPK-PDPIRRAVLRAIEVGYRHFDTAAHYET---EAPIGEAAA-EAVRSGAVASRDDLFITSKLWC 82
OsJ_deoxymugineic_ac ---------MSDGGAGAKGAGFGMPRVGMGTAVQGPR-PEPIRRAVLKAIEAGYRHFDTAAHYET---EAPIGEAAA-EAVRSGAIASRADPFITSKLWC 86
Zm_Deoxymugineic_aci -------------MSATGRAPCGLPRVGLGTAVQGPR-PDPVRAAVLRAIQLGYRHFDTAAHYAT---EAPIGEAAA-EAVRTGLVASREDLFVTSKVWC 82
Pap_som_NADPH-depend -------MESNGVPMITLSSGIRMPALGMGTAETMVKGTEREKLAFLKAIEVGYRHFDTAAAYQS---EECLGEAIA-EALQLGLIKSRDELFITSKLWC 89
Gm_NAD_P_H_dependent ---MAAAIEIPTIVFPNSSAQQRMPVVGMGSAPDFTC-KKDTKEAIIEAVKQGYRHFDTAAAYGS---EQALGEALK-EAIHLGLVS-RQDLFVTSKLWV 91
PsP_Morphine_6-dehyd --------MAGKSPLINLNNGVKMPALGLGVFAASAE---ETASAIASAISSGYRLIDTARSYNN---EAQVGEGIR-NSG-----VDRAEMFVTTKLFN 80
At_quinone_oxidoredu -MTATNKQVILKDYVSGFPTESDFDFTTTTVELRVPEGTNSVLVKNLYLSCDPYMRIRMGKPDPS---TAALAQAYTPGQPIQGYGVSRIIESGHPDYKK 96
At_AER               -MTATNKQVILKDYVSGFPTESDFDFTTTTVELRVPEGTNSVLVKNLYLSCDPYMRIRMGKPDPS---TAALAQAYTPGQPIQGYGVSRIIESGHPDYKK 96
At_K+_channel_protei ---------MQYKNLGKSGLKVSTLSFGAWVTFGNQLDVKEAKSILQCCRDHGVNFFDNAEVYANGRAEEIMGQAIRELGWRRSDIVISTKIFWGGPGPN 91
Ps_AKR1__igrA_       --------MHREDDSTSTGRREERLSTGKGDSLQPGPDQRIFQTALYRPQRHGLILFPQR-LYGNCLAPLAYSNYVAPRELHSSIHAEGRTLSVRLIMLF 91

At_Aldo-keto_reducta TD-LDPPDVQDALNRTLQDLQLDYVDLYLMHWPVRLKKGTVDFKPE--NIMPIDIPSTWKAMEALVDSGKARAIG------------VSNFSTKKLSDLV 169
At_Aldo-keto_reducta TY-HDPQEVPEALNRTLQDLQLDYVDLYLIHWPVSLKKGSTGFKPE--NILPTDIPSTWKAMESLFDSGKARAIG------------VSNFSSKKLADLL 169
Dp_Aldose_reductase  TD-HAPEDVPVALDKTLEDLQLDYIDLYLIHWPVRLKKGSVGLDPE--NFVPTDIPGTWKAMEALYDSGKARAIG------------VSNFTLKKLSDLL 169
Zm_F352_Aldose_reduc DH-HAPEDVPEALNDSLNDLQLEYLDLYLIHWPFRVKKG-TNTSPE--NFVTPDFPATWGAMEKLYDAGKARAIG------------VSNFSSKKLGDLL 165
Ha_AR__ADR           TN-LAPERVRPALENTLKDLQLDYIDLYHIHWPFRLKDGAHMPPEAG-EVLEFDMEGVWKEMENLVKDGLVKDIG------------VCNYTVTKLNRLL 177
Hv_Deoxymugineic_aci SD-AHGDRVVPALRHTLRNLQMEYVDLYLVHWPVSMKPGRFKAPFTAEDFVPFDMRAVWEAMEECHRLGLAKAIG------------VANFSCKKLDTLL 169
Ta_Deoxymugineic_aci SD-AHRDRVVPALRQTLRNLQMEYVDLYLVHWPVSMKPGRFKAPFTADDFVPFDMRAVWEAMEECHRLGLAKAIG------------VANFSCKKLDTLL 169
OsJ_deoxymugineic_ac SD-AHRDRVLPALRQTLWNLQMEYVDLYLVHWPVSMKPGRYKAPFTADDFVPFDMRAVWEAMEECHRLGLAKAIG------------VCNFSCKKLDTLL 173
Zm_Deoxymugineic_aci AD-AHRDRVLPALRRTLSNLQMEYVDLYMVHWPVTMKAGRFTAPFTPEDFEPFDMRAVWEAMEECHRLGLAKAIG------------VCNFSCKKLETLL 169
Pap_som_NADPH-depend AD-AHADLVLPALQNSLRNLKLEYLDLYLIHHPVSLKPGKFVNEIPKDHILPMDYKSVWAAMEECQTLGFTRAIG------------VSNFSCKKLQELM 176
Gm_NAD_P_H_dependent TE-NHPHLVLPALRKSLKTLQLEYLDLYLIHWPLSSQPGKFSFPIEVEDLLPFDVKGVWESMEECQKLGLTKAIG------------VSNFSVKKLQNLL 178
PsP_Morphine_6-dehyd CD-YGYERALRAFDESLGRLGLDYVDLYLLHWPTK---------------DWNATIQSWKAAEKILGDGRARAIG------------VCNFLEDQLDELI 152
At_quinone_oxidoredu GD-LLWGIVAWEEYSVITPMTHAHFKIQHTDVPLSYYTGLLGMPGMT-AYAGFYEVCSPKEGETVYVSAASGAVGQL----------VGQLAKMMGCYVV 184
At_AER               GD-LLWGIVAWEEYSVITPMTHAHFKIQHTDVPLSYYTGLLGMPGMT-AYAGFYEVCSPKEGETVYVSAASGAVGQL----------VGQLAKMMGCYVV 184
At_K+_channel_protei DKGLSRKHIVEGTKASLKRLDMDYVDVLYCHRPDASTPIEEAVRAMN--YVIDKGWAFYWGISEWSAQQITEAWG-----------------AADRLDLV 172
Ps_AKR1__igrA_       VNTSKRSRTLPPVGSTVMRILVNLCQLLSAEFQLLQGSQRLIFKLCYRTCPDQRRGDTRVAQCPGDSHLSQCLTATFRNGVQRPDAPQQLFVLCTGLQGA 191

At_Aldo-keto_reducta EAARVPPAVNQVECHPSWQQHKLHEFCKSKGIHLSGYSPLGSPG------TTWVKADVLKSPVIEMIAKEIGKSPAQTALRWGLQMGHSILPKSTNEGRI 263
At_Aldo-keto_reducta VVARVPPAVNQVECHPSWQQNVLRDFCKSKGVHLSGYSPLGSPG------TTWLTSDVLKNPILGGVAEKLGKTPAQVALRWGLQMGQSVLPKSTHEDRI 263
Dp_Aldose_reductase  DVARIPPAVNQVGCHPSCAQTKLRAFCKSKGIHLSGYSPLGSPG------TPWVKHDVLENPILVDVAEKLGKTPAQVALRWGLQMGHSVLPKSVHESRI 263
Zm_F352_Aldose_reduc AVARVPPAVDQVECHPGWQQTKLHSFCQSTGVHLTAYSPLGSPG------TTWMNGNVLKEPIIISIAEKLGKTSAQVALRWNIQMGHSVLPKSTNEERI 259
Ha_AR__ADR           RSAKIPPAVCQMEMHPGWKNDKIFEACKKHGIHVTAYSPLGSS-----------EKNLAHDPVVEKVANKLNKTPGQVLIKWALQRGTSVIPKSSKDERI 266
Hv_Deoxymugineic_aci SFATIPPTVNQVEVNPVWQQRKLREFCRGKGIQLCAYSPLGAKG------THWGSDAVMDAGVLQDIAASRGKSVAQVCLRWVYEQGDCLIVKSFDEARM 263
Ta_Deoxymugineic_aci SFATIPPTVNQVEVNPVWQQRKLREFCRGKGIQLCAYSPLGAKG------THRGSDAVMDAGVLQEIAASRGKSVAQVCLRWVYEQGDCLIVKSFDEARM 263
OsJ_deoxymugineic_ac SFATIPPAVNQVEVNPVWQQRKLRELCREKGVQICAYSPLGASG------THWGSDSVMASAVLRDIAQSKGKTVAQVCLRWVYEQGDCLIVKSFDEARM 267
Zm_Deoxymugineic_aci SFATIPPVVNQVEINPVWQQRKLREFCRAKGIQLCAYSPLGAKG------THWGSDSVMDSGVLHEIAKSKGKTVAQVCLRWVYEQGDCLIVKSFDEGRM 263
Pap_som_NADPH-depend AAAKIPPVVNQVEMSPTLHQKNLREYCKANNIMITAHSVLGAIG------APWGSNAVMDSKVLHQIAVARGKSVAQVSMRWVYQQGASLVVKSFNEGRM 270
Gm_NAD_P_H_dependent SVATIRPVVDQVEMNLAWQQKKLREFCKENGIIVTAFSPLRKG-------ASRGPNEVMENDVLKEIAEAHGKSIAQVSLRWLYEQGVTFVPKSYDKERM 271
PsP_Morphine_6-dehyd AASDVVPAVNQIELHPYFAQKPLLAKNRALGIVTEAWSPIGGAINDGDGDNHGGRKHPLTDPVITTIAEAHGRSAAQVILRWHFQN--DVIPKSVNPERI 250
At_quinone_oxidoredu GSAGSKEKVDLLKTKFGFDDAFNYKEESDLTAALKRCFTNGIDIY----FENVGGKMLDAVLVNMNMHGRIAVCGMISQYNLENQEGVHNLSNIIYKRIR 280
At_AER               GSAGSKEKVDLLKTKFGFDDAFNYKEESDLTAALKRCFPNGIDIY----FENVGGKMLDAVLVNMNMHGRIAVCGMISQYNLENQEGVHNLSNIIYKRIR 280
At_K+_channel_protei GPIVEQPEYNMFARHKVET--EFLPLYTNHGIGLTTWSPLASG-------VLTGKYNKGAIPSDSRFALENYKNLANRSLVDDVLRKVSGLKPIAGELGV 263
Ps_AKR1__igrA_       ALIRPRPFGDSVQIAGGQQSLSQRRKDNAAGSDLAKGIEQAIFDP-AIEHVVIGLMNEKRNPLFLQDRGSLLRQFRRIAGNPHIKRLALTVQMRERSHRL 290

At_Aldo-keto_reducta REN---FDVLGWSIPKEMFDKFSKIEQAR--LVQGTSFVHETLSPYKTLEELWDGEI----------- 315
At_Aldo-keto_reducta KQN---FDVFNWSIPEDMLSKFSEIGQGR--LVRGMSFVHETS-PYKSLEELWDGEI----------- 314
Dp_Aldose_reductase  KEN---IDVFSWCIPDVLFAKFSEIEQVS--PGKPEFPVHPEISQYKTVEEMWDGGI----------- 315
Zm_F352_Aldose_reduc KQN---LDVYDWSIPDDLLAKFSEIKQAR--LLRGNFIVNPES-VYKTHEELWDGEL----------- 310
Ha_AR__ADR           KEN---IQVFGWEIPEEDFKVLCSIKDEKRVLTGEELFVNKTHGPYRSAADVWDHEN----------- 320
Hv_Deoxymugineic_aci REN---LDVDGWELTEEERRRIAEIPQRK---INLGKRYVSDHGPYKSLEELWDGEI----------- 314
Ta_Deoxymugineic_aci REN---LDVDGWELTEEERRRIAEIPQRK---INLGKRYVSEHGPYKSLEELWDGEI----------- 314
OsJ_deoxymugineic_ac REN---LDIVGWELTEEERQRIAGIPQRK---INRALRFVSDHGPYKSLDDLWDGEI----------- 318
Zm_Deoxymugineic_aci KEN---LDIVDWELSEEERQRISKIPQRK---INQGRRYVSEHGPYKSFEELWAGEI----------- 314
Pap_som_NADPH-depend KEN---LKIFDWELTAEDMEKISEIPQSR---TSSAAFLLSPTGPFKTEEEFWDEKD----------- 321
Gm_NAD_P_H_dependent NQN---LHIFDWALTEQDHHKISQISQSR---LISG-------PTKPQLADLWDDQI----------- 315
PsP_Morphine_6-dehyd AKN---IDVFDFALSDAEMAQLDELDTGVR------IGPDPRDVDTSSFAEFV--------------- 294
At_quinone_oxidoredu IQG---FVVSDFYDKYSKFLEFVLPHIREGKITYVEDVADGLEKAPEALVGLFHGKNVGKQVVVVARE 345
At_AER               IQG---FVVSDFYDKYSKFLEFVLPHIREGKITYVEDVADGLEKAPEALVGLFHGKNVGKQVVVVARE 345
At_K+_channel_protei TLA---QLAIAWCASNPNVSSVITGATRGSQIQENMKAVDVIPLLTPIVLDKIEQVIQSKPKRPESYR 328
Ps_AKR1__igrA_       FQRRGGVHTVRIEDVDIVEPHPLQRPGRGWRSGICDLPPMPPYGPGHMSQPAFEEMIISSR------- 351
```

Figure S2. Homology of PsAKR1 with other characterized proteins: Homology of PsAKR1 with other characterized AKR proteins. The sequences were aligned using SMS2.0 (sequence manipulation tool) and conserved regions at amino acid level for co-factor binding and substrate binding was highlighted. The highlighted regions at 28 (G), 30 (G), 62 (Y), 93 (N), 106 (T), 117 (L), 182 (F), 198 (P), 200 (D), 224 (L), 244 (G), 272 (R), 289 (R), 292 (Q) and 242 (F) amino acids are conserved among the AKR family of genes.

```
OsAKR1  ------------------------------------------------------------ 0
OsALR1  ------------------------------------------------------MDSTLK 6
PsAKR1  MHREDDSTSTGRREERLSTGKGDSLQPGPDQRIFQTALYRPQRHGLILFPQRLYGNCLAP 60

OsAKR1  ---------MQIHWPFRVK---------KGSGISNTEDYIPPDIPSTWG----------A 32
OsALR1  DLQLDYVDLYLIHWPFQIK---------KGTELS-PENFVKPDIPSTWR----------A 46
PsAKR1  LAYSNYVAPRELHSSIHAEGRTLSVRLIMLFVNTSKRSRTLPPVGSTVMRILVNLCQLLS 120

OsAKR1  MEKLYDSGKSRAIGVSNFSSKK---LGDLLAVACVP------------------------ 65
OsALR1  MEQLYDSGKARAIGVSNFSSKK---LGDLLCVARVP------------------------ 79
PsAKR1  AEFQLLQGSQRLIFKLCYRTCPDQRRGDTRVAQCPGDSHLSQCLTATFRNGVQRPDAPQQ 180

OsAKR1  -----------------PAVDQVECHPGWQQTKLHNFCQSTGVHLSVSLWYLYNYSESML 108
OsALR1  -----------------PAVDQVECHPGWQQAKLRAFCHTSGVHLSAYAPLGRMKGIAVD 122
PsAKR1  LFVLCTGLQGAALIRPRPFGDSVQIAGGQQSLSQRRKDNAAGSDLAKGIEQAIFDPAIEH 240

OsAKR1  LLMSPVVFN----FLNT------------ANPFLPLLFRHTRL----------------- 135
OsALR1  SVLPSVAEM----LGRTPAQVALRWGLQQGQSVLPKSVSEARLKENMDLFGWSIPEELCA 178
PsAKR1  VVIGLMNEKRNPLFLQDRGSLLRQFRRIAGNPHIKRLALTVQMRERSHRLFQRRGGVHTV 300

OsAKR1  ---------------------------------------------------          135
OsALR1  KLSEIEQVK----QIRGDGFAHPESVYKTYEELFDGEI-------------          212
PsAKR1  RIEDVDIVEPHPLQRPGRGWRSGICDLPPMPPYGPGHMSQPAFEEMIISSR          351
```

Figure S3. Homology of PsAKR1 with OsAKR1 and OsALR1 : The conserved regions at amino acid level for cofactor binding and substrate binding was highlighted. We aligned the sequences using SMS2.0 (sequence manipulation tool)(Stothard 2000) . In rice there are ~27 AKR like proteins, and to study the effectiveness of highly homologous to PsAKR1 protein that is OsAKR1 was selected and also another AKR family protein OsALR1 selected because their target substrates may vary with the presence of substrate carbonyl compound.

a)

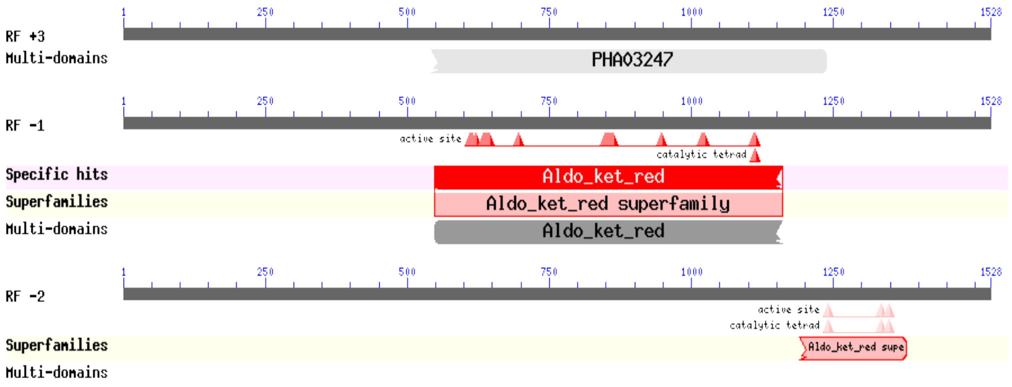

List of domain hits	PssmId	Multi-dom	E-value
PHA03247[PHA03247], large tegument protein UL36; Provisional	223021	yes	2.39e-05
Aldo_ket_red[cd06660], Aldo-keto reductases (AKRs) are a superfamily of soluble NAD(P)(H) oxidoreductases whose ...	119408	no	4.16e-50
Aldo_ket_red[pfam00248], Aldo/keto reductase family; This family includes a number of K+ ion channel beta chain regulatory domains - these are ...	215817	yes	4.25e-48
Aldo_ket_red super family[cl00470], Aldo-keto reductases (AKRs) are a superfamily of soluble NAD(P)(H) oxidoreductases whose ...	241886	no	1.70e-30

b)

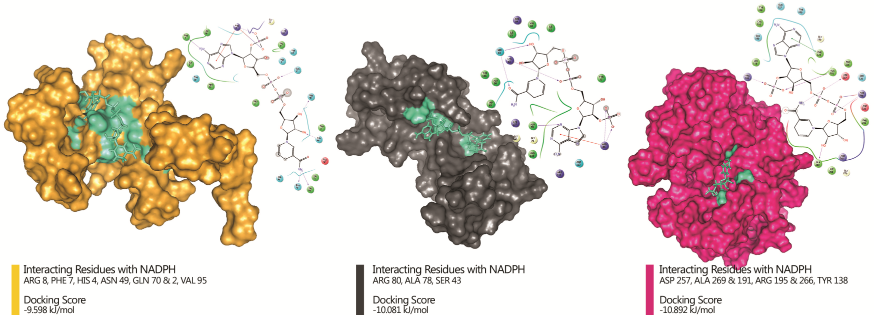

Figure S4. Conserved domains prediction on PsAKR1 protein and molecular docking of AKR proteins with co-factor NADPH : a)The conserved domains were predicted using CDDv3.10-44354 PSSMs database. http://www.ncbi.nlm.nih.gov/Structure/cdd/wrpsb.cgi. PsAKR1(igrA) Graphical summary and list of different domain hits. b) Molecular docking of primary phase of OsAKR*1*, OsALR1 and PsAKR1 proteins with co-factor NADPH. i) 2D and 3D interactive map of OsAKR1 with NADPH complex, ii) OsALR1 with NADPH, iii) PsAKR1 with NADPH.

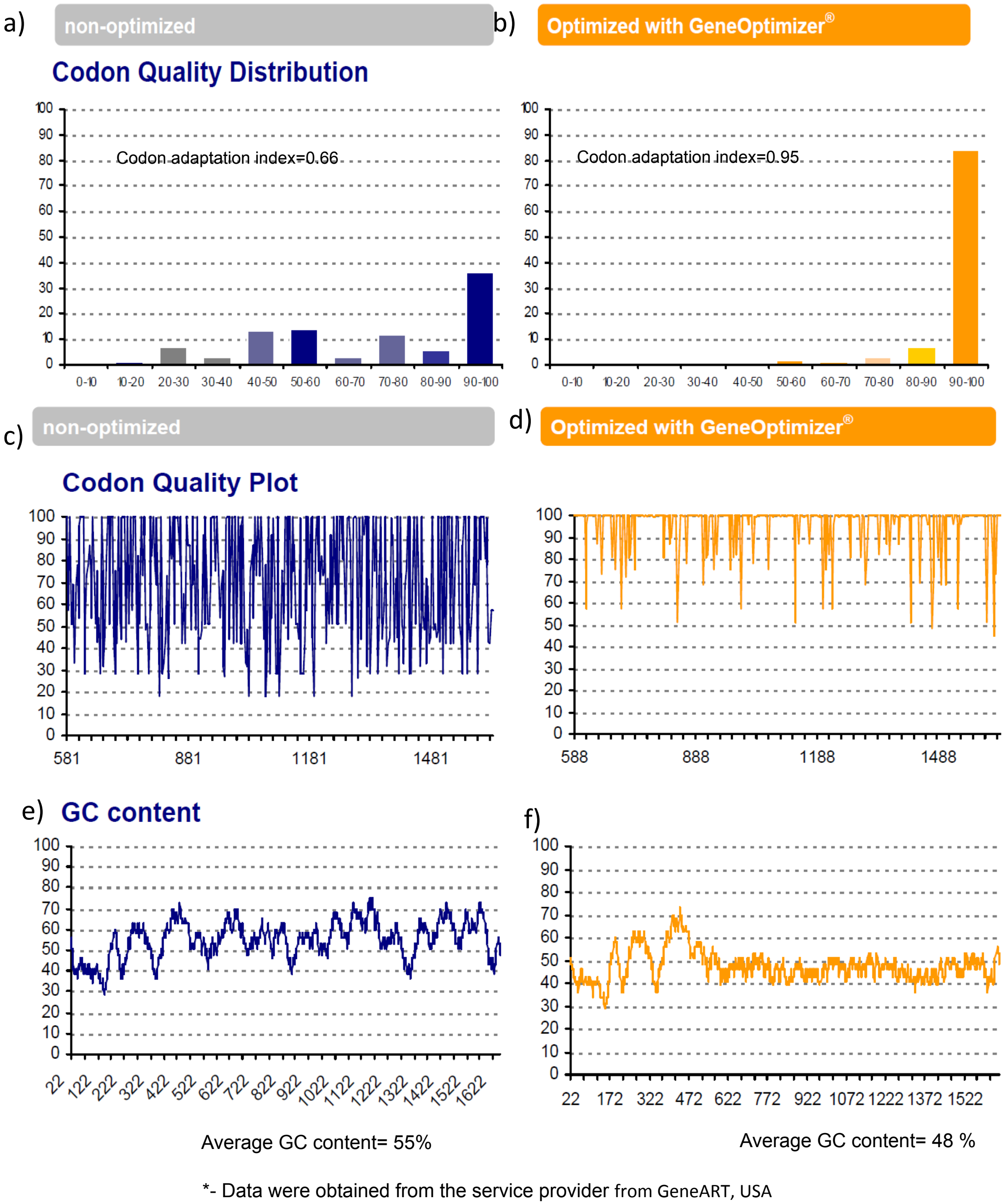
a)
non-optimized
b)
Optimized with GeneOptimizer®
Codon Quality Distribution
Codon adaptation index=0.66
Codon adaptation index=0.95
0-10 10-20 20-30 30-40 40-50 50-60 60-70 70-80 80-90 90-100
c)
non-optimized
d)
Optimized with GeneOptimizer®
Codon Quality Plot
581 881 1181 1481
588 888 1188 1488
e)
GC content
f)
22 122 222 322 422 522 622 722 822 922 1022 1122 1222 1322 1422 1522 1622
22 172 322 472 622 772 922 1072 1222 1372 1522
Average GC content= 55%
Average GC content= 48 %
*- Data were obtained from the service provider from GeneART, USA

Figure S5.

Figure S5. Codon optimization of *PsAKR1* gene to plants. a&b) The histograms show the percentage of sequence codons, which fall into a certain quality class. The quality value of the most often used codon for a given aminoacid in the desired expression system is set to 100, the remaining codons are scaled accordingly (See also Sharp, P.M., Li, W.H., Nucleic Acids Res. 15 (3), 1987). c&d) The plots show the quality of the used codon at the indicated codon position. e&f) The plots show the GC content in a 40bp window centered at the indicated nucleotide position

The Pseudomonas strain pg2982 tolerant to glyphosate and gene responsible for resistance have been cloned and characterized in *E.Coli*. It is been known fact that the bacterial genes are AT rich and optimization requires to express in eukaryotic systems. In this context the *igrA* (*PsAKR1*) gene (Acc. No. M37389) from pseudomonas was codon optimized. The codon usage was adapted to the codon bias for *dicots*. In addition, regions of very high (> 80%) or very low (< 30%) GC content have been avoided where possible. During the optimization process cis-acting sequence motifs were avoided including internal TATA-boxes, chi-sites and ribosomal entry sites, AT-rich or GC-rich sequence stretches, RNA instability elements ("Killer motifs"), repeat sequences and RNA secondary structures, (cryptic) splice donor and acceptor sites, branch points, Two STOP codons were added to ensure efficient termination. The Increased GC-content is known to prolong mRNA-half life and integrity in plants. However, increasing the GC content has somehow negative influence on the codon usage in dicots. The *PsAKR1* gene was carefully designed and balanced between codon usage on the one hand and increasing GC-content on the other hand. The optimization was successfully carried out with no negative cis-acting sites (such as splice sites, poly(A) signals, etc) which may negatively influence expression. Codon usage was adapted to the bias of *dicots* resulting in a high CAI value (0.95). (CAI: The parameter CAI (codon adaptation index) describes how well the codons match the codon usage preference of the target organism. Thus, a CAI of 1.0 would be perfect. However, a CAI of > 0.9 is considered as very good (i.e. allowing high expression). In addition to the codon optimization to improve the expression levels the polyA (AAAAAAAAAA) signal sequence was added upstream to the igrA gene. The recruitment of the 43S pre-initiation complex to an mRNA is a competitive process that is determined by features of the mRNA. The mechanism of initiation of translation in plants and other eukaryotes involves a pseudo-circularized mRNA formed by interactions between the 5'-cap and 3'-tail which involve eIF4G, eIF4B and poly (A) binding protein. The modified consensus sequence was characterized by a preference for A from position 10 to 1. This is favored in highly translated mRNAs is consistent with the analyses of AUG context in stably transformed cells. So we have added these poly A signal (10nt) upstream to the gene

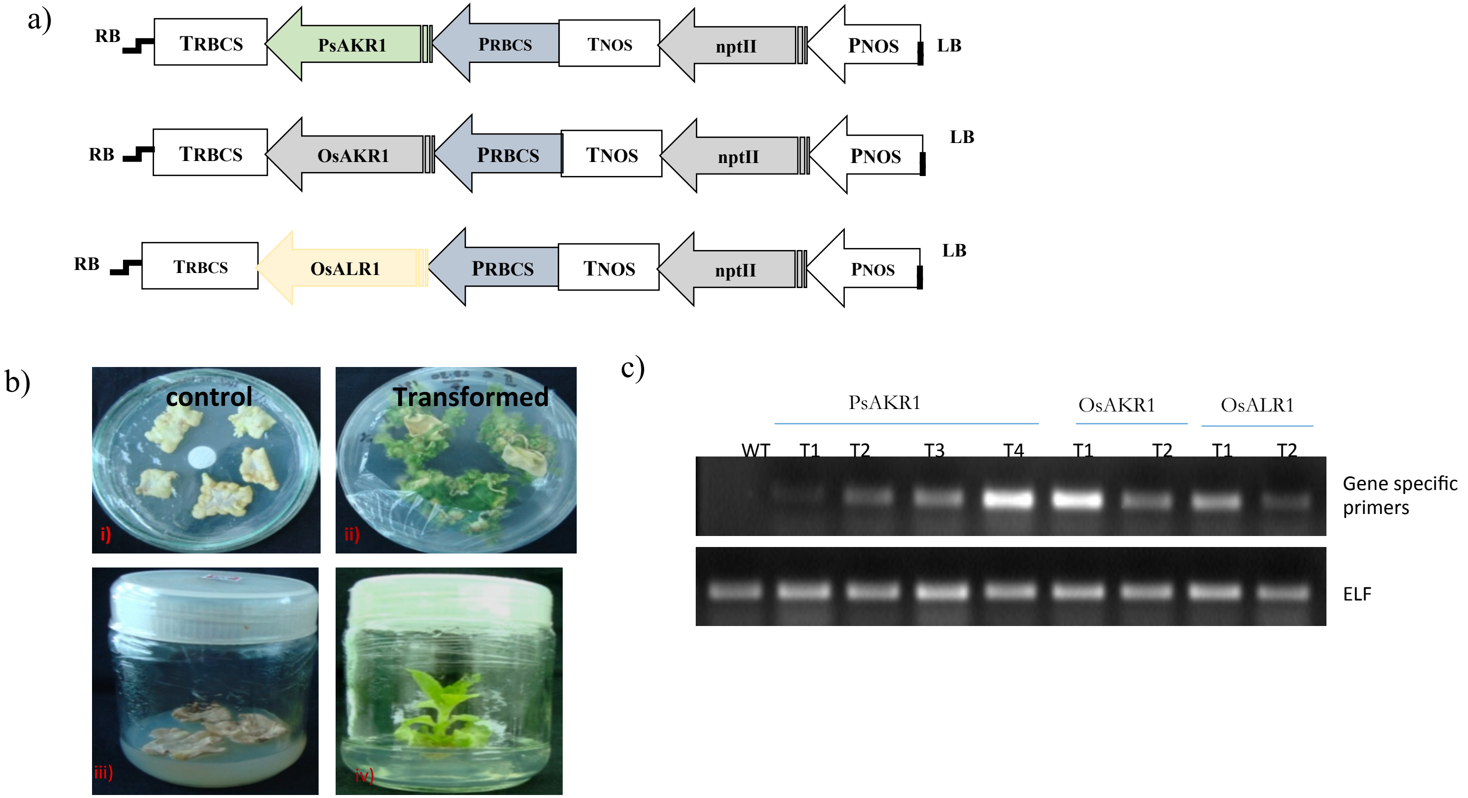

Figure S6. Regeneration of tobacco explants transformed with *AKR* and *OsALR1* gene constructs. a) Schematic representation of gene constructs developed in Binary vector pBINplus, b) Regeneration and transformation of *RBCS::PsAKR1* tobacco leaf explants on glyphosate medium. i) &ii) Regeneration of wild-type explants without *AKR1* (control) and with *AKR1* (transformed) on MS media containing 0.012 mg/ml of glyphosate, iii) & iv) Selection of *PsAKR1* infected explants and plantlets grown on glyphosate (0.012 mg/ml) containing MS media, c) Molecular analysis using. Semi-quantitative RT-PCR to confirm the expression of genes. The transgenic plants were developed and the expression was confirmed in T1 generation by isolating RNA from individual plants. These plants were used for glyphosate resistant assays.

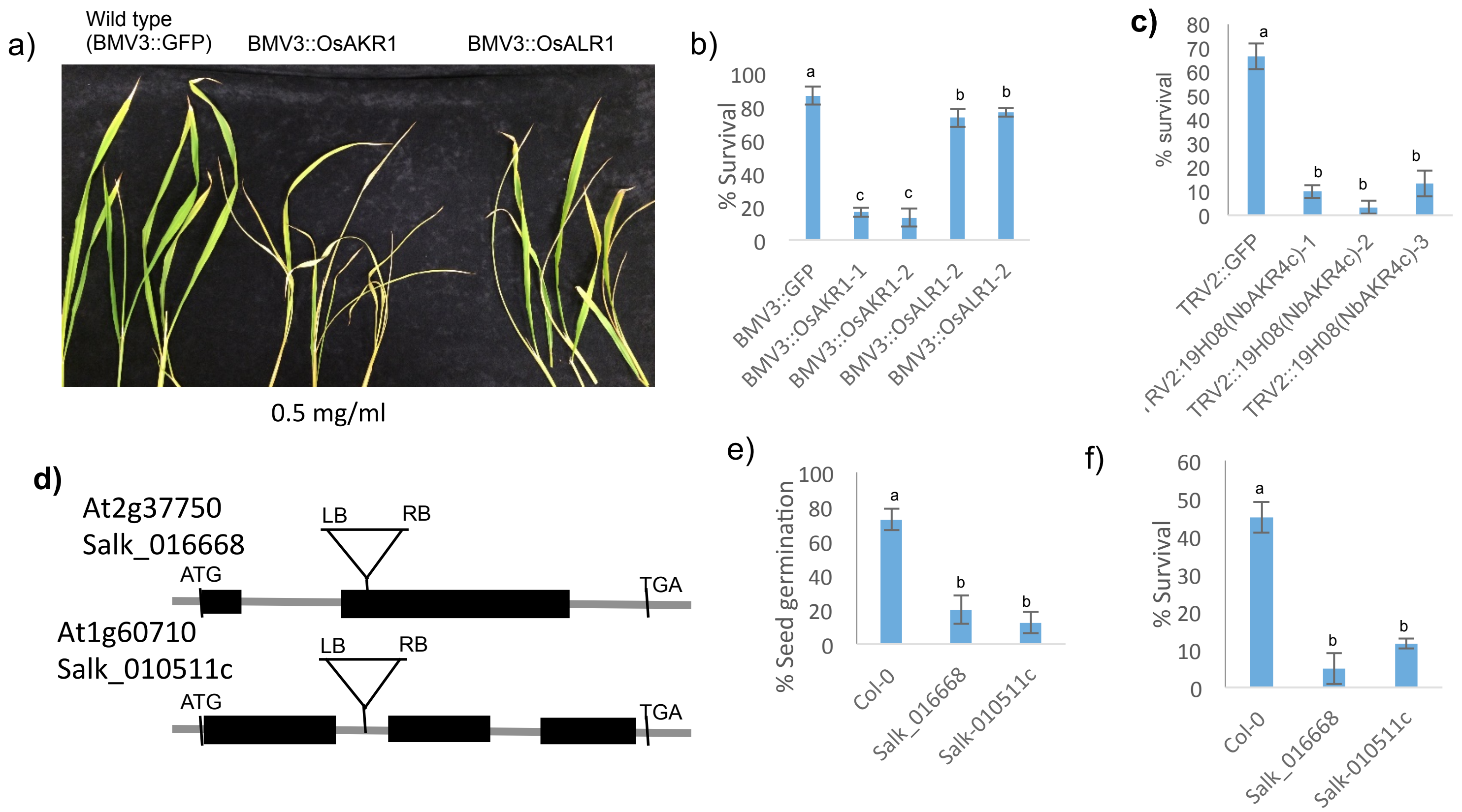

Figure S7. Response of Os*AKR1* and Os*ALR1* silenced rice, *N.benthamiana* and Arabidopsis plants against glyphosate. a) Photograph showing the effect of glyphosate (0.5 mg/ml) on rice seedling, b) Seedling survival rate after glyphosate spray. 4-week old *OsAKR1* and *OsALR1* silenced rice seedlings were sprayed with 0.5 mg/ml of glyphosate and after one week the survival rate was recorded. The results from two way ANOVA with Tukey's HSD means separation test (α= 0.05), are presented. Same letters are not statistically significantly different. C) Surival rate of N.benthamiana NbAKR4c VIGS silinced plants silenced after glyphosate spraying . D-f) Characterization of *Arabidopsis AKR* homolog mutants. Structure of *Arabidopsis* At2g37750 *and* At1g60710 genes with the T-DNA insertion. Black boxes indicate exons, and gray lines represent noncoding regions. The triangles show the T-DNA positions, e) Germination response to glyphosate- the seeds were planted on ½ MS media containing 0.02 mg/ml of glyphosate and percent of seed germination was recorded after 6 days, f) Survial rate of mutant Arabidopsis plants after glyphosate spraying.

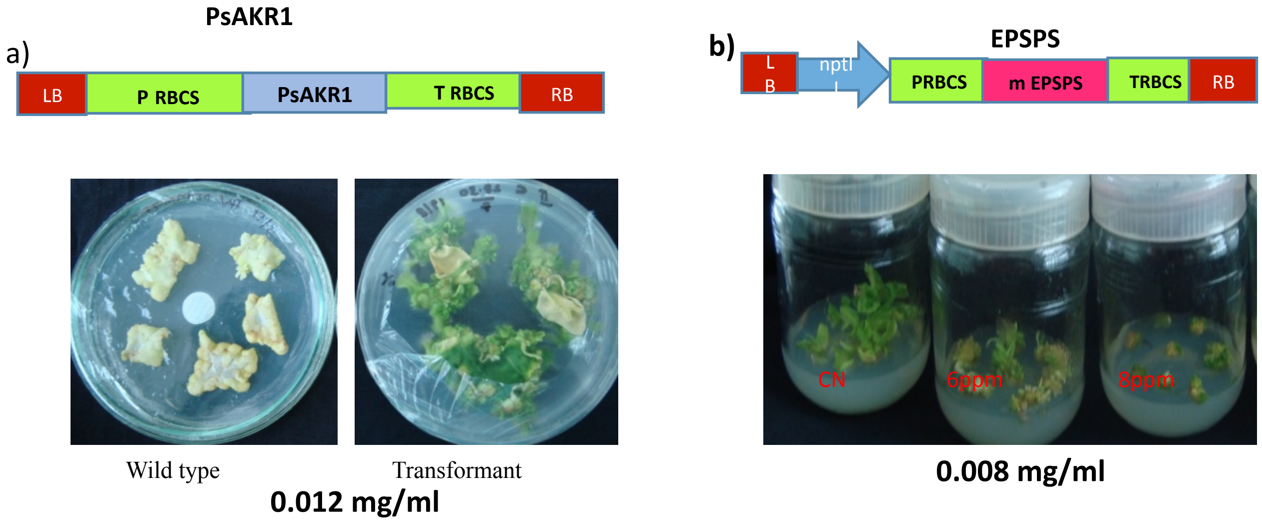

Figure S8. Regeneration efficiency of *PsAKR1* and *mEPSPS* expressing transgenic plants. a)The *PsAKR1* expressing explants could regenerate even up to 0.012 mg/ml of glyphosate, b) *mEPSPS* expressing explants could regenerate only upto 0.008 mg/ml of glyphosate, The leaf discs from transgenic tobacco expressing *PsAKR1* and *mEPSPS* plants were regenerated on different concentrations of glyphosate media. Minimum of 25 leaf discs from each transgenic plants were used to asses in 3 different experiments.

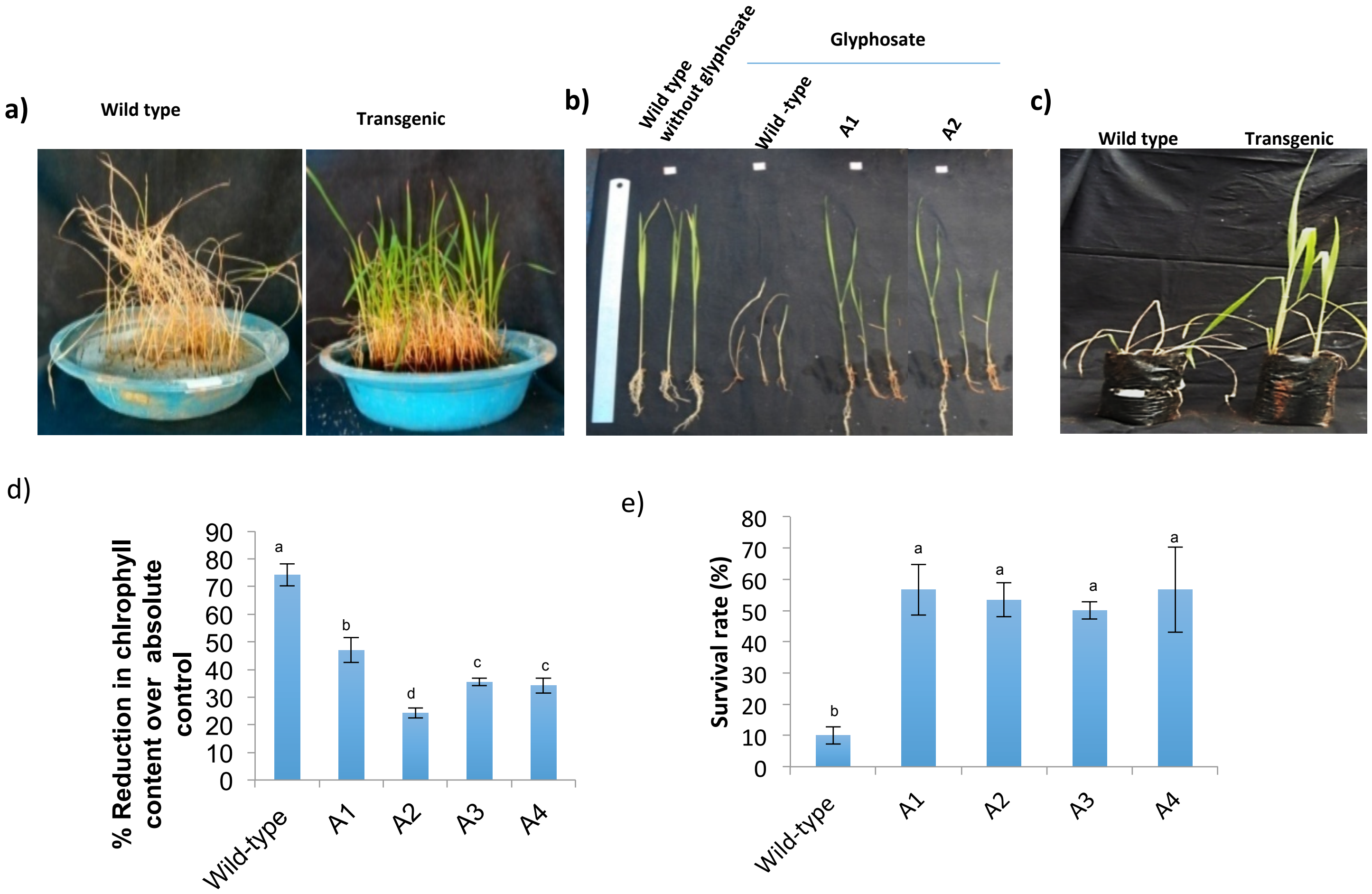

Figure S9. Response of rice transgenic plants expressing *PsAKR1* on glyphosate. A) Screening of rice transformants on 0.025 mg/ml of glyphosate in T1 generation- The pre-germinated seeds were transformed to sterilized quartz sand containing 0.025 mg/ml of glyphosate for 10 days to asses the glyphosate resistance, b) photographs of survived seedlings after 10 days, c) Response of transgenic rice plants to leaf swabbing assay- matured green leaves from established plants was swabbed with 2 mg/ml of glyphosate and maintained in green house conditions until the wild type seedlings showing complete wilting phenotype as shown in figure. Only *PsAKR1* expressing transgenic plants were survived, d) Chlorophyll content from the rice transgenic plants treated with 2 mg/ml of glyphosate, e) Survival rate of transgenic seedlings. Minimum of 25 seedlings from each transgenic lines were used to asses glyphosate resistance. Experiment was repeated for three times. The results from two way ANOVA with Tukey's HSD means separation test (α= 0.05), are presented. Same letters are not statistically significantly different.

a)

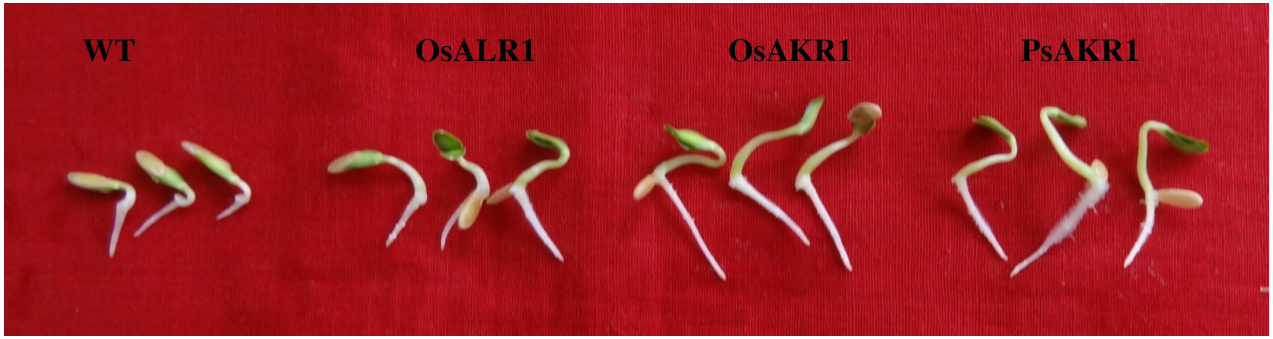

b)

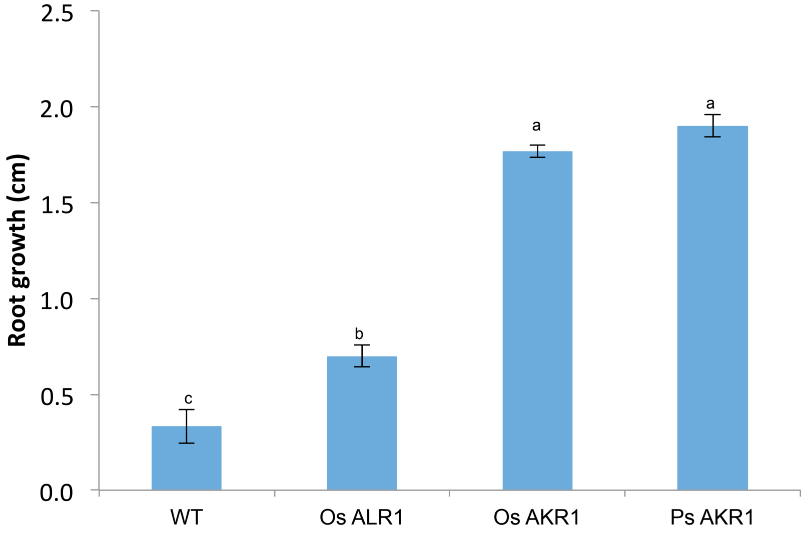

Figure S10. Degradation of glyphosate by AKR proteins from plant. The crude proteins were extracted from leaves expressing PsAKR1, OsAKR1, OsALR1 and wild types tobacco plants and incubated with 500 ppm of glyphosate for 1 h. The glyphosate degradation was assessed by cucumber seedling growth. **a**) Growth of cucumber seedlings with PsAKR1, OsAKR1 proteins did not inhibited by glyphosate whereas OsALR1 and wild type leaf proteins showed significant inhibition, b) Root growth of cucumber seedlings with PsAKR1 and OsAKR1 proteins is significantly higher compared to OsALR1 and WT proteisn confirming AKRs specifically degrade glyphosate. Minimum of 25 seedlings were treated with 10 μg of crude proteins mix and 0.5 mg/ml glyphosate. The results from two way ANOVA with Tukey's HSD means separation test (α= 0.05), are presented. Same letters are not statistically significantly different.
