# Supplementary material for: Aldo‐keto reductase enzymes detoxify glyphosate and improve herbicide resistance in plants
Source: Plant Biotechnol J. 2017 May 11;15(7):794–804. doi: 10.1111/pbi.12632 (PMC5466437; doi:10.1111/pbi.12632)
Supplement: Supplementary file 2 — Table S1 Screening and segregation analysis of the transgenic tobacco expressing PsAKR1, OsAKR1 and OsALR1 plants in T1 generation. Table S2 Lethal dose (LD) determination of tobacco explants. Table S3 Gene constructs used in this study. Table S4 Gene fragments used to silence N. benthamiana AKR and OsAKR1 and OsALR1 genes in rice. Table S5 Primers used in this study. [file PBI-15-794-s003.docx]

**Table S1. Screening and segregation analysis of the transgenic tobacco expressing *PsAKR1*, *OsAKR1* and *OsALR1* plants in T1 generation.**

| Line | 0.2 mg/ml of Glyphosate | | |  |
| --- | --- | --- | --- | --- |
|  | Total number of seeds | Glyphosate positive/ tolerant seedling | Glyphosate negative /number of bleached seedlings | chi square |
| PsAKR1-T1 | 90 | 70 | 20 | 0.37037 |
| PsAKR1-T2 | 115 | 90 | 25 | 0.652174 |
| PsAKR1-T3 | 103 | 81 | 22 | 0.728155 |
| PsAKR1-T4 | 99 | 72 | 17 | 0.91746 |
| OsAKR1-T1 | 105 | 80 | 25 | 0.079365 |
| OsAKR1-T2 | 98 | 75 | 23 | 0.122449 |
| OsLAR1-T1* | 96 | 75 | 21 | 1.817869 |
| OsALR1-T2* | 110 | 75 | 35 | 0.981818 |
| WT | 110 | 7 | 103 | 276.3758 |

The transgenic plants showing 3:1 ratio of segregation based on resistance were considered as homozygous lines and were used for further experiments. The seeds were grown on 0.2 mg/ml of glyphosate media. In case of *OsALR1* the seedlings were screened on Kanamycin (100mM). The chi-square test and segregation pattern was calculated as described in James et al., 2002.

* - The *OsALR1* transformed plants were not survived on glyphosate. However, to get transgenic plants for further comparative studies, *OsALR1* expressing transgenic plants were developed on kanamycin selection medium.

**Table S2. Lethal dose (LD) determination of tobacco explants.**

| Glyphosate concentration in mg/l | Standardization of lethal dose in Wild type explants | *Agrobaterium* harbouring empty binary vector | *Agrobacteriu*m strain EHA105 harbouring binary vector with *PsAKR1* |
| --- | --- | --- | --- |
| 0 | 25 | 25 | 25 |
| 1 | 25 | 25 | 25 |
| 2 | 25 | 24 | 25 |
| 4 | 23 | 22 | 25 |
| 6 | 8 | 8 | 25 |
| 8 | 1 | 1 | 25 |
| 10 | 0 | 0 | 20 |
| 12 | 0 | 0 | 16 |
| 15 | 0 | 0 | 8 |
| 20 | 0 | 0 | 2 |

Two independent experiments were carried out in tobacco leaves from cv KST-19, In experiment-I, 15 explants were maintained and in experiment –II, 25 explants were maintained to determine the LD value of glyphosate for tobacco regeneration medium and presented here. The direct regeneration of tobacco leaf explant was used to standardize lethal dose of glyphosate and explants transformed with *PsAKR1* construct expressing Agrobacterium culture. The non-infected wild-type explants were cultured on different concentrations of glyphosate to identify lethal dose at which 50% of explants die. At 0.008 mg/ml glyphosate concentration, tobacco explants did not survive. Based on this information *PsAKR1* expressing explants were transferred to MS agar plates with 0.012 mg/ml of glyphosate (Supplementary Fig. 8c). *PsAKR1* expressing explants showed significantly improved regeneration efficiency and few explants survived even at 0.02 mg/ml of glyphosate. The regeneration efficiency clearly demonstrates that PsAKR1 can detoxify glyphosate efficiently. A total of 35 plants were generated and all the T0 plants could set seeds without showing any abnormal phenotypic variation. The T1 generation plants were screened against glyphosate and identified homozygous lines and used in subsequent experiments.

**Table S3. Gene constructs used in this study.**

| sl.no | construct | Vector |
| --- | --- | --- |
| 1 | pRBCS::*PsAKR1*:tRBCS | pBINplus |
| 2 | pRBCS::*OsAKR1*:tRBCS | pBINplus |
| 3 | pRBCS::*OsALR1*:tRBCS | pBINplus |
| 4 | 2x35SCaMV:*:mEPSPS*:tNos | pBINAR |
| 5 | T7 promoter::*PsAKR1*:T7 | pET-32a(+) |
| 6 | T7 promoter::OsAKR1:T7 | pET-32a(+) |
| 7 | T7 promoter::OsALR1:T7 | pET-32a(+) |
| 8 | TRV2::NbAKR | pQVIET2 |
| 9 |  | TRV1 |
| 10 |  | BMV-RNA1 |
| 11 |  | BMV-RNA2 |
| 12 | BMV::OsAKR1 | BMV-RNA3 |
| 13 | BMV::OsALR1 | BMV-RNA3 |

**Table S4. Gene fragments used to silence N.*benthamiana* AKR and *OsAKR1* and *OsALR1* genes in rice.**

| Gene fragment | Sequence |
| --- | --- |
| *NbME19H08- AKR* | tgattctgaaatgtacaacatggcttaaaggtgatgtgctaaagcagccagctgtagtctcagttgctgagaaattaggcaagactcctgctctggtttgtcttcgttggggcattcgatgatttatttgccaagttctctgaaattccacaggcaaggctgcttagaggcacttcatttgttcacgaggcttatggccagtaaatgggtcagagtgttcttcctaagagcacacatgaagcacggatcaaagagaatctwgatgtattggactggtctatacc |
| *OsAKR1* | atgcagattcattggccattcagagtcaagaagggctcaggcattagtaacactgaagactacataccacctgacatcccatctacctggggagcaatggagaagctatatgattctggtaaatctcgtgccattggtgtaagtaacttctcatcaaaaaaactgggtgacctgcttgctgtagcctgtgtacctccagctgttgatcaggtagaatgccatcctggttggcagcaaacgaagctacataacttctgccagtcaactggcgttcatctttctgtaagtctgtggtacttat |
| *OsALR1* | ggtgcagcgacctcgcacctgaagatgtcccactcgcaatggatagtacactgaaagatctgcagctggattacgttgatctgtatctgattcattggccgtttcagatcaagaaaggcacggagctcagtccagagaacttcgtcaagcctgacatacccagcacctggcgagcgatggagcagctgtacgattcaggcaaagctcgcgcgatcggcgtgagcaatttctcctccaagaagcttggcgatctgctctgcgtcgcccgcgtccctccggccgttgatcaggtcgagtgcc |

**Table S5. Primers used in this study**

| Sl.no | primer name | sequence |
| --- | --- | --- |
| 1 | PsAKR1- F | GATGCACAGAGAGGACGATAGC |
| 2 | PsAKR1-R | ATCAAAGCTGCGCCTTGTAGC |
| 3 | OsAKR1-F | GGGCCATGGGTCCCGTAGTCTCGTGGC |
| 4 | OsAKR1-R | GCGGCCGCAGACAAACAAGTAGCATGG |
| 5 | OsALR1-F | GGGCCATGGGACTACTGATTTTCCCTTCG |
| 6 | OsALR1-R | GCGGCCGCGTGAAATGCCTGTTATT |
| 7 | PsAKR1qRT-F | CCGGCAACCCTCACATCAAAAGATTGGC |
| 8 | PsAKR1qRT-R | GGAGGCATTGGAGGCAGGTCACAGATTCC |
| 9 | OsAKR1qRT-F | CCTGCACAAGTGGCACTGCACTGG |
| 10 | OsAKR1qRT-R | GCAGACAAACAAGTAGCATGG |
| 11 | OsALR1qRT-F | ATGGGACTACTGATTTTCCCTTCGBottom of Form |
| 12 | OsALR1qRT-R | GCTCAGGTTGCTCTGCGCTGG |
| 13 | mEPSPS –RT-F | CAGGGTTTTCAGAAACGAGACC |
| 14 | mEPSPS-RT-R | ACTGCTTTCCCATTGGTTGCTG |
| 15 | pET-OsAKR F | GGGCCATGGCGAAGCATTTCGTGCAAC |
| 16 | pET-OsAKR R | GCTCGAGTCTAAATTTCGCCGTCCCAGA |
| 17 | pET-Os ALR F | GGGCCATGGATAGTACACTGAAAGATC |
| 18 | pET-OsALR1 R | GGCTCGAGTTAGATTTCTCCATCAAAAAG |
| 19 | Nb.Actin-qRT-F | CGGAATCCACGAGACTACATAC |
| 20 | Nb.Actin-qRT-R | GGGAAGCCAAGATAGAGC |
| 21 | At.actin-qRT-F | Top of Form  CTCAGGTATTGCAGACCGTATGAGBottom of Form  Bottom of Form |
| 22 | At.actin-qRT-R | Top of Form  CTGGACCTGCTTCATCATACTCTG Bottom of Form |
| 23 | Top of Form  At1g60710 -qRTRBottom of Form | GCACCCTTTCTTCTCCGATATT Bottom of Form |
| 24 | Top of Form  At1g60710-qRTFBottom of Form | GGATGACTTCAGAAAGGCTCTAC Bottom of Form |
| 25 | Top of Form  At2g37750-qRTRBottom of Form | GACGCCCTATTGGAGGAATTAG Bottom of Form |
| 26 | Top of Form  At2g37750-qRTF Bottom of Form | CTTCTTACTACGTTCGTCTTCCCBottom of Form |
| 27 | NbME Top of Form  19H08 -qRTR Bottom of Form | CCATAAGCCTCGTGAACAAATG Bottom of Form |
| 28 | NbME Top of Form  19H08 -qRTFBottom of Form | CACATGAAGCACGGATCAAAG Bottom of Form |
| 29 | Top of Form  OsALR1BMV RBottom of Form | GGCCATGGGGCACTCGACCTGATCAACGGBottom of Form |
| 30 | Top of Form  OsALR1BMV FBottom of Form | GGCCTAGGGGTGCAGCGACCTCGCACCTG Bottom of Form |
| 31 | Top of Form  OsAKR1BMV RBottom of Form | GGCCATGGTAAGTACCACAGACTTACAGA Bottom of Form |
| 32 | Top of Form  OsAKR1BMV FBottom of Form | GGCCTAGGATGCAGATTCATTGGCCATTC Bottom of Form |
| 3 | Os18sRNA-qRTF | Top of Form  GAATTCCTAGTAAGCGCGAGTCAT Bottom of Form |
| 30 | Os18sRNA-qRTR | Top of Form  CAATGATCCTTCCGCAGGTTC Bottom of Form |
