# Supplementary material for: Aldo‐keto reductase enzymes detoxify glyphosate and improve herbicide resistance in plants
Source: Plant Biotechnol J. 2017 May 11;15(7):794–804. doi: 10.1111/pbi.12632 (PMC5466437; doi:10.1111/pbi.12632)
Supplement: Supplementary file 3 — Appendix S1 Materials and methods related to vector construction and Modeling of OsAKR1, PsAKR1 and OsALR1 structure. [file PBI-15-794-s001.docx]

Supplementary information- I

**Materials and methods related to vector construction and Modeling of OsAKR1, PsAKR1 and OsALR1 structure**

**Vector construction –** The codon optimized *PsAKR1* gene was cloned into impact vector 1.1 ([www.pri.org/impact vector](http://www.pri.org/impact%20vector)) driven by RBCS promoter and RBCS terminator at *BamHI* and *SmaI* sites. Further, the whole gene cassette was cloned into *pBINplus* binary vector using *AscI*, *PacI* restriction enzymes. The *pBINplus pRBCS:: PsAKR1:tRBCS* construct was mobilized to *Agrobacterium* strain *EHA105* through electroporation using Eppendorf’s electroporation model with 1,440 volts for 5 milliseconds. Similarly *OsAKR1* and *OsALR1* genes were obtained from RIKEN research center (JAPAN) (Seki *et al.* 2002) and cloned into impact vector and subsequently to *pBINplus* and mobilized to *Agrobacterium* strain *EHA105 (*Supplementary Table 3*)*. The rice VIGS constructs were prepared by using Brome mosaic virus (BMV) by amplifying the *OsAKR1* and *OsALR1* gene fragments (Supplementary Table 4) with *NcoI* and *AvrI* restriction endonucleases flanking primers (Supplementary Table 5). For silencing in N. benthamiana TRV2-VIGS clone NbME19H08 (Supplementary Table 4) encoding an aldo-keto reductase was identified from the VIGS library (<http://vigs.noble.org/line2.php?id=NbME19H08>).

**Modeling of** **OsAKR1, PsAKR1 and OsALR1 structure:** The structures of OsAKR1, PsAKR1 and OsALR1 were hierarchically modeled and validated. Homomultimer methods were incorporated from Schrodinger prime to model proteins wherein energy based analysis was consumed to align the template correspondingly with OsAKR1, PsAKR1 and OsALR1 [1]. Template PDB structures were aligned to correspond domain region with OsAKR1 (PDB ID: 4GAC, 4FZI, 4IJC and 3H7U), OsALR1 (PDB ID: 4Q3M 1PZ1 3H7U) and PsAKR1 (PDB ID: 1X1Q, 2H3J) (Berman *et al.,* 2000). The threshold limit was set up for centile and maintained greater than 60% of similarity matching with subject. The total consensus similarity of OsAKR1, PsAKR1 and OsALR1 were conserved as 88%, 84.5% and 82.5%, respectively.

Validation of modeled OsAKR1, PsAKR1 and OsALR1 structure: *Phase I Validation*- The modeled proteins were subjected to plot against Ramachandran's conformation Library (φ and ψ backbone dihedral angles library) (Sastry *et al.,* 2013). There are four categories of classified plotted amino acids in the Ramachandrans plot such as most favored region (A,B,L), additional favored region [a,b,p,l], generously allowed region (-a,-b,-p,-l) and disallowed region (Laskowski *et al.,* 2001). *Phase II Validation-* The modeled proteins were set up to minimize from its current energy by a module from Schrodinger-Macromodel. So the energies such as Stretch Energy (strE), Bend Energy (benE), Torsion Energy (torE), Improper Torsion Energy (impE), vDW Energy (vDWE), Electrostatic Energy (elecE) and Solvation Energy (solE) were calculated for native form of modeled protein and post minimized form of modeled protein. Both current and minimized energy Parameter log includes the force field of OPLS 2010, water as solvent used, charges based on force field, van der Waals up to 8 angstrom and H Bond up to 2 angstrom. The Parameter log information was set based on the size and volume of OsAKR1, OsALR1 and PsAKR1 structure. *Phase III Validation |* The modeled proteins were submitted to Structural Analysis and VeR1fication Server (SAVES) to find the cumulative root-mean-square deviation (rmsD) of each proteins aminoacid conformation and to find average Z Score. To acknowledge the range of submitted protein, rms and Z score values were enabled as regression points (Pontius *et al.,* 1996)

**Docking of** **OsAKR1, PsAKR1 and OsALR1 structure with NADPH and glyphosate:** Phase I (Target Protein with NADPH) and Phase II (Target Protein + NADPH + Glyphosate) type of molecular docking were performed to advocate the profile of functional complex. Profiled information and energy calculation of post-interacted complex defines the binding efficiency of each protein.

*Phase I Docking of Target protein with NADPH:* Modeled 3D structure of OsAKR1, PsAKR1 and OsALR1 proteins were interacted with NADPH (Compound Identifier no: 52945042) (Kim et al., 2016) by glide module from Schrodinger suite. Since the target protein has no destination of choosing active site specificity because of redox potential nature of NADPH/NADP^+^ (Schrödinger Release 2015), the docking procedure was preferred to have Flexible and Grid points selected as maximum of X [60A], Y [60], Z [60] in three dimensional coordinates (Cho *et al.,* 2005). The van der Waals scaling factor of each protein was set up to have 1.0 and charge cut off as 0.25. This signifies the inclusion of nonpolar parts of the protein as absolute value. Docking log profile prepared to have extra precision descriptions information which gives the G score (Glide) value as the addition of H bond, energy of vdw, energy of coulomb, energy of rotational bond, energy of model complex, internal energy, ligand efficiency and ligand efficiency of surface accessible values (Cho et al., 2005).

*Phase II docking of target proteins with NADPH and glyphosate:* To initiate second phase of Molecular Docking, the analysis of finding higher binding affinity of NADPH specificity with OsAKR1, OsALR1 and PsAKR1 was progressed. The complex of PsAKR1 with NADPH, OsAKR1 with NADPH and PsAKR1 with NADPH structures were subjected to interact with Glyphosate [Compound Identifier no: 3496] with same attributes as phase I molecular docking profile. The resulting complexes were analyzed on ranges of glide score obtained from extra precision property description profile.

**Molecular dynamics simulations of OsAKR1, PsAKR1 with NADPH and glyphosate complex for 10 ns:** The complex structures were allowed to simulate to find structural stability of the complex with glyphosate. GROMOS96 force field proffered to simulate under explicit simple point charge from Gromacs Package. Complexes were soaked with TIP3P water layer as orthorhombic surface because of the calculations of surface accessible solvent volume. Solvent profile was set up to have a 3D boundary as 90A, 90A, 90A for rotatable group and maximum distance of 10A, 10A, 10A. Internal quality of the models was solvated with the explicit dynamics with the periodic boundary conditions (PBC) and energy minimization to the system that concerns Steepest Decent Method. 10 nanoseconds of MD simulation were set up to find 1,000 trajectories from 10 ps interval time. The epoch of individual complexes were recognized to be more with glyphosate. 310 K temperature kept as Berenson's temperature coupling algorithm. Particle mesh Ewald algorithm was computed to calculate electrostatic interactions. Internal bond energy calculated by linear constraint solver algorithm.

**Expression analysis**

Total RNA was extracted according to the protocol described by Datta *et al.* (1989) and first strand cDNA was synthesized by oligo (dT) primers using Molony Murine Leukaemia Virus reverse transcriptase (MMLV-RT; MBI Fermentas, Hanover, MD, USA) according to manufacturer’s instructions. The cDNA pool was used as a template to perform RT- PCR analysis. PCR conditions were 94°C for 2 minutes, 25 cycles of 94°C for 45 seconds, 52-58°C for 30 seconds, 72°C for 30 seconds and a final extension of 72°C for 10 minutes. The quantitative real-time RT-PCR was performed with the fluorescent dye SYBR-green (TAKARA SYBR-green qPCR Kit) following the manufacturer’s protocol (Opticon2, MJ research, USA). The conditions for the PCR were as follows: 95ºC for 2 minutes, 25 cycles of denaturation at 94ºC for 45 seconds, annealing for 30 seconds (56ºC and 58ºC for genes and elongation factor, respectively), polymerization for 45 seconds (72ºC) followed by plate reading at 72ºC for 5 minutes, estimation of melting curve from 50ºC to 95ºC and incubation at 72ºC for 4 minutes. The relative expression levels of the selected genes under a given stress condition was calculated using comparative threshold method (Supplementary Table 5).

**MDA (lipid peroxidation) estimation –** The glyphosate-treated leaf tissue was used for measuring MDA levels. About 0.5-1.0 g of tissue was homogenized in 5 mL of 5% (w/v) trichloroacetic acid and the homogenate was centrifuged at 10,000 RPM for 15 min at room temperature. The supernatant was mixed with an equal volume of thiobarbituric acid [0.5% in 20% (w/v) trichloroacetic acid], and the mixture was boiled for 25 minutes at 100°C, followed by centrifugation for 5 minutes at 10,000 RPM to clarify the solution. Absorbance of the supernatant was measured at 532 nm and corrected for nonspecific turbidity by subtracting the A600. MDA content in leaf tissue was calculated using standard graph (Loreto and Velikova et al., 2001).

**Photosynthetic rate measurements:** 6-week-old (50 day-old) tobacco transgenic plants were maintained in pots under greenhouse conditions. 1 mg/ml of glyphosate was sprayed to both wild-type and transgenic tobacco plants. Effect of glyphosate on photosynthetic machinery was studied four days after spraying. Gas exchange parameters such as photosynthetic rate (μmolm^-2^s^-1^) and stomatal conductance (mmol^-2^s^-1^) were measured using a portable photosynthetic system using LICOR 6400, Lincoln, Nebraska, USA (Loreto and Velikova et al., 2001). The plants were assessed for dry matter accumulation by weighing stem and leaves biomass.

### **Estimation of total chlorophyll content**

Chlorophyll was extracted from 500 mg of leaf tissue in acetone:DMSO (1:1) and total volume made up 25ml. The absorbance was recorded at 663 nm and 645 nm using UV-visible spectrophotometer (UV 2450, Shimadzu Corporation, and Kyoto, Japan). Total chlorophyll was expressed as mg/g FW using the following formula (Babitha *et al.*, 2015)

For, CHLa=[12.7(A_663_)-2.69(A_645_)×V]/(W×1000),

For CHLb=[22.9(A_645_)-4.68(A_663_) ×V]/(w×1000) and

Total chlorophyll, TCHL(mg/gFW)= CHLa+CHLb

Where, CHL = chlorophyll, V = volume, W = weight and TCHL = Total chlorophyll

**References**

Babitha, K.C., Vemanna, R.S., Nataraja, K.N. & Udayakumar, M. (2015) Overexpression of EcbHLH57 transcription factor from eleusine coracana l. in tobacco confers tolerance to salt, oxidative and drought stress. *Plos one* ***10,*** *e0137098*.

Berman, H.M., Westbrook, J., Feng, Z., Gilliland, G., Bhat, T.N., Weissig, H., Shindyalov, I.N., Bourne, P.E. (2000) The Protein Data Bank. [*Nuc. Acids Res*. 28, 235-242](http://nar.oxfordjournals.org/cgi/content/abstract/28/1/235)

Cho, A. E., Guallar, V., Berne, B., Friesner, R. A. (2005) Importance of Accurate Charges in Molecular Docking: Quantum Mechanical/Molecular Mechanical (QM/MM) Approach. [*J. Comput. Chem.* *26*, 915–931](http://www3.interscience.wiley.com/cgi-bin/abstract/110472845/ABSTRACT)

Datta, S.K., Patel, H. & Berry, D. (1989) Extraction and purification of RNA from crop plants. *J. Exp. Bot.* **165**, 1252.

Kim, S., Thiessen, P.A., Bolton, E.E., Chen, J., Fu, G., Gindulyte, A., et al. (2016) PubChem Substance and Compound databases. Nucleic Acids Res. 44, D1202–D1213. doi: 10.1093/nar/gkv951

Laskowski R. A, MacArthur M. W, Thornton J. M. (2001) PROCHECK: validation of protein structure coordinates, in *International Tables of Crystallography, Volume F. Crystallography of Biological Macromolecules*, eds. Rossmann M G & Arnold E, Dordrecht, Kluwer Academic Publishers, The Netherlands, 722-725

Loreto, F. & Velikova, V. (2001) Isoprene produced by leaves protects the photosynthetic apparatus against ozone damage, quenches ozone products and reduces lipid peroxidation of cellular membranes. *Plant Physiol.* **127,** 1781-1787.

Pontius J, Richelle J, Wodak S.J. (1996) Deviations from standard atomic volumes as a quality measure for protein crystal structures. J Mol Biol. 264, 121-36.

Sastry, G.M., Adzhigirey, M., Day, T., Annabhimoju, R., Sherman, W. (2013) Protein and ligand preparation: Parameters, protocols, and influence on virtual screening enR1chments.  [*J. Comput. Aid. Mol. Des.* *27*, 221-234](http://link.springer.com/article/10.1007/s10822-013-9644-8).

Schrödinger Release 2015-4 (2015): LigPrep, version 3.6, Schrödinger, LLC, New York, NY.

Seki, M., Narusaka, M., Kamiya, A., Ishida, J., Satou, M. *et al.* Functional annotation of a full-length Arabidopsis cDNA collection. Science, 296, 141–145 (2002)
